# Efficacy and Safety of Rotigotine Transdermal Patch on Neuropsychiatric Symptoms of Parkinson's Disease: An Updated Meta-Analysis and Systematic Review

**DOI:** 10.3389/fneur.2021.722892

**Published:** 2021-10-21

**Authors:** Junqiang Yan, Hongxia Ma, Anran Liu, Jiarui Huang, Jiannan Wu, Jianxue Yang

**Affiliations:** ^1^Key Laboratory of Neuromolecular Biology, The First Affiliated Hospital, College of Clinical Medicine of Henan University of Science and Technology, Luoyang, China; ^2^Department of Neurology, The First Affiliated Hospital, College of Clinical Medicine of Henan University of Science and Technology, Luoyang, China; ^3^School of Nursing, The First Affiliated Hospital of Henan University of Science and Technology, Luoyang, China

**Keywords:** rotigotine transdermal patch, Parkinson's disease, neuropsychiatric symptoms, meta-analysis, systematic review

## Abstract

**Objective:** The effects of rotigotine transdermal patch (RTG) on the neuropsychiatric symptoms of Parkinson's disease (PD) outcomes remain controversial. The aim of this review was to determine the efficacy and safety of RTG on the neuropsychiatric symptoms of PD.

**Methods:** In this systematic review and meta-analysis, PubMed, Cochrane Library, EMBASE, and Web of Science were searched for randomized controlled trials comparing RTG and placebo in PD up to May 10, 2021. We analyzed the data using Review Manager 5.2 software. The quality of evidence was assessed using the Grading of Recommendations Assessment, Development, and Evaluation Approach. In order to avoid false-positive results caused by random error, we use TSA software for trial sequential analysis (TSA).

**Results:** We included 10 studies (1,844 patients). The meta-analysis showed that, compared with placebo, RTG can significantly improve the scores for Apathy Scale (MD = −1.68, 95% confidence interval, CI: −2.74 to −0.62, *P* = 0.002; moderate certainty), Beck Depression Inventory-II (MD = −1.19, 95% CI: −2.26 to −0.11, *P* = 0.03; moderate certainty), the Non-Motor Symptoms Scale (MD = −3. 66, 95% CI: −4. 30 to −3.01, *P* < 0.00001; moderate certainty), the sleep/fatigue domains of the Parkinson's Disease Non-motor Symptom Assessment Scale (MD = −2.03, 95% CI: −3.08 to −0.98, *P* = 0.0001; moderate certainty), the mood/apathy domains of the Non-motor Symptom Scale (MD = −2.48, 95% CI: −4.07 to −0.89, *P* = 0.002; high certainty), the eight-item Parkinson's Disease Questionnaire (MD = −4. 93, 95% CI: −6.79 to −3.07, *P* < 0.00001; moderate certainty), and the 39-item Parkinson's Disease Questionnaire (MD = −3.52, 95% CI: −5.25 to −1.79, *P* < 0.0001; high certainty). However, there was no statistically significant difference on the Snaith–Hamilton Pleasure Scale (MD = −0.12, 95% CI: −0.58 to 0.34, *P* = 0.61). Our results showed that RTG exerts a positive effect on sleep. According to the TSA, the results implied that, except for the Beck Depression Inventory-II, conclusive evidence have been obtained in the RTG group. It has been proven in our meta-analysis that rotigotine has good safety and tolerability.

**Conclusions:** RTG can effectively improve the neuropsychiatric symptoms, sleep quality, and quality of life in patients with PD.

## Introduction

Parkinson's disease (PD) is the second most common progressive neurodegenerative disorder characterized by motor and non-motor symptoms. Although PD is considered a typical movement disorder, more than 90% of PD patients suffer from non-motor symptoms, such as emotional disorders and apathy, sleep disorders, and depression, and these non-motor symptoms seriously affect the quality of life of patients with PD (1, 2).

There is currently no specific medication for PD. Rotigotine, a non-ergolinic D_3_/D_2_ and D_1_-dopamine agonist, is suitable for transdermal delivery *via* skin patches that contain the drug in a silicone-based adhesive matrix, and its efficacy can last more than 24 h with a daily patch application (3, 4). The previous meta-analysis showed that RTG can effectively improve the neuropsychiatric symptoms in patients with PD (5), but a recently published randomized controlled trial showed that there was no statistically significant difference between RTG and placebo in the treatment of neuropsychiatric symptoms of PD (6). To assess the efficacy and safety of RTG in the treatment of neuropsychiatric symptoms of PD, we updated the previous meta-analysis.

## Methods

### Search Strategy

Published relevant studies were searched in PubMed, Cochrane Library, EMBASE, and Web of Science up to May 10, 2021. The keywords used for standard searches included “Parkinson's disease,” “rotigotine,” and “randomized controlled trials.” Titles and abstracts were independently screened by two authors. We take PubMed as an example, and the detailed search strategy is presented in Supplementary Material 1.

### Inclusion and Exclusion Criteria

The inclusion criteria were as follows: (1) study type: randomized controlled trial (RCT), (2) participants: the patients were clinically diagnosed with PD, (3) interventions: the experimental group was given a rotigotine transdermal patch, and the control group was given a placebo, and (4) outcome: at least one of the 10 instruments was employed, including the eight-item Parkinson's Disease Questionnaire (PDQ-8), Beck Depression Inventory (BDI-II), Parkinson's Disease Non-motor Symptom Assessment Scale (NMSS), Parkinson's Disease Sleep Scale (PDSS), the Modified Parkinson's Disease Sleep Scale (PDSS-2), Snaith–Hamilton Pleasure Scale (SHAPS), the 39-item Parkinson's Disease questionnaire (PDQ-39), Apathy Scale (AS), Pittsburgh Sleep Quality Index (PSQI), and polysomnography (PSG) sleep parameters.

The exclusion criteria were as follows: (1) non-randomized controlled trial, (2) repeated publication, and (3) studies whose outcome measures were not reported.

### Data Extraction

The following data were collected from each included study: (1) baseline characteristics, including the author, year of publication, study design, age, sample size, sex ratio, duration of treatment, measuring tools, Hoehn and Yahr, and duration of PD; (2) mean change of PDQ-8 score, BDI-II score, NMSS total score, sleep/fatigue score of NMSS, mood/apathy score of NMSS, PDSS score, PDSS-2 score, SHAPS score, PDQ-39 score, AS score, PSQI score, and PSG sleep parameters from baseline to the end of treatment. Mean and standard deviation (SD) were extracted whenever possible. We calculated the SD using standard error (SE) when only SE was reported; and (3) the number of adverse events, including application site reactions, nausea, dizziness, headache, insomnia, fatigue, and dyskinesia.

### Assessment of Risk of Bias

We used the Cochrane Risk of Bias Tool to assess the risk of bias. The assessment tool is composed of seven parts, including random sequence generation, allocation consultation, blinding of the participants and personnel, blinding of outcome assessment, incomplete outcome data, selective reporting, and other biases. The assessment results were shown as “low risk of bias,” “high risk of bias,” or “unclear risk of bias.” The risk of bias of each included randomized controlled trial was assessed independently by two authors, and another author resolved the disagreement.

### Trial Sequential Analysis

Due to data sparseness and repeated testing of accumulated data, the accumulated meta-analysis has the risk of producing random errors, so we conducted the analysis using Trial Sequential Analysis, v. 0.9.5.10 beta software (Copenhagen Trial Unit, Center for Clinical Intervention Research, Rigshospitalet, Copenhagen, Denmark, https://www.ctu.dk/tsa) (7, 8). TSA forms a boundary value curve by correcting random errors, which is called the TSA boundary value. The horizontal line of *Z* = 1.96 is the traditional significant horizontal line (α = 0.05), which is called the traditional boundary value. In order to minimize random errors, we calculated the required information size (the number of patients needed) in a meta-analysis to detect or reject a certain intervention effect (8). In our meta-analysis, estimating the required information size was based on a type I error of 5%, a type II error of 20%, and the expected mean difference and variation which was estimated by combined meta-analysis in the included trials with a low risk of bias. We applied a model variance-based heterogeneity correction. When the accumulated *Z*-value of the meta-analysis crosses the traditional boundary value before the required information size is reached, but does not cross the TSA boundary value, it means that the traditional meta-analysis may have a false-positive conclusion and means that more trials need to be included to confirm the efficacy. When the accumulated *Z*-value of meta-analysis crosses both the TSA boundary value and the traditional boundary value before the required information size is reached, this means that conclusive evidence may have been established, and further trials may not be required. When the accumulated *Z*-value of the meta-analysis neither crosses the traditional boundary value nor the TSA boundary value before the required information size is reached, there may be no statistical difference between the intervention group and the control group in efficacy, and more trials are still needed.

### Rating Quality of Evidence

The Grading of Recommendations Assessment, Development, and Evaluation (GRADE) can offer a system for the rating quality of evidence in systematic reviews and the grading strength of recommendations in guidelines (9). We use GRADEpro software (Evidence Prime Incorporation, McMaster University; https://gradepro.org) to rate the quality of evidence on a certain outcome. In the GRADE process, RCTs start as high-quality evidence, and five factors which include risk of bias, inconsistency, indirectness, imprecision, and publication bias can lead to rating down the quality of evidence. Observational studies start as low-quality evidence, and three factors which include large effects, plausible confounding changing the effect, and dose–response grade may lead to rating up the quality of evidence (9). Finally, the quality of evidence for each outcome is rated as one of high quality, moderate quality, low quality, and very low quality. Two investigators conducted qualitative discussions on each item of GRADE, and a final verdict on a certain body of evidence was obtained. Any disagreement would be settled by another author. The results were presented as the GRADE evidence profile.

### Statistical Analyses

Review Manager (RevMan 5. 2) was used to conduct this meta-analysis. We calculated the odds ratio (OR) among the data of dichotomous variables and calculated the mean difference (MD) among the data of continuous variables. Furthermore, 95% confidence interval (CI) was used to represent each effect size. In the results, a *p*-value of < 0.05 was considered statistically significant. We estimated the heterogeneity between studies using *I*^2^ statistics. When *I*^2^ <50%, there was no significant heterogeneity in the included studies, and a fixed-effects model was applied. When *I*^2^ ≥ 50%, there was heterogeneity, and a random-effects model was applied. Next, we used sensitivity analysis to detect the sources of heterogeneity. The sensitivity analysis was conducted using Stata SE software (STATA 16.0; https://www.stata.com/).

## Results

### Literature Search and Study Characteristics

**Figure 1:**
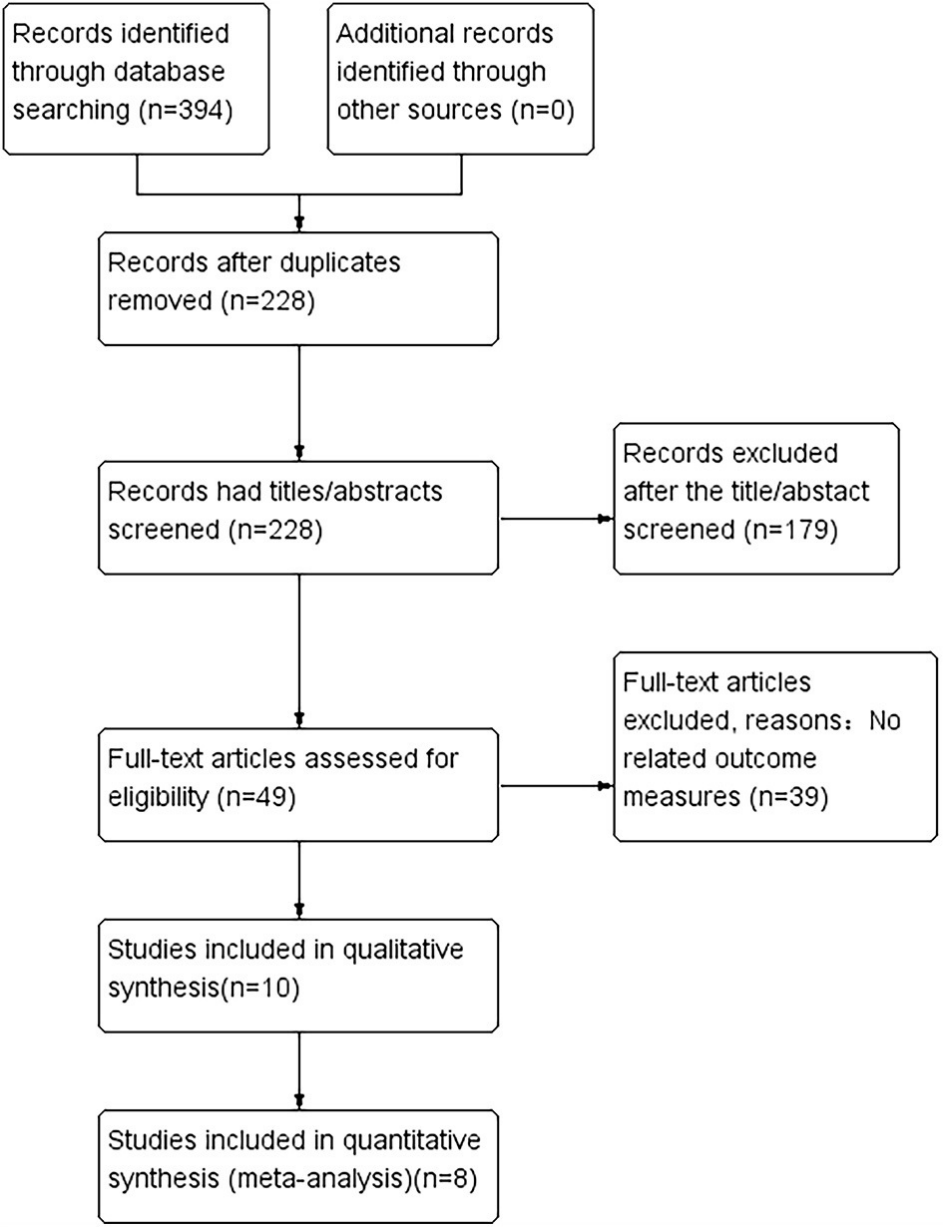
Study flow diagram.

As shown in Figure 1, we identified 394 records from PubMed, Cochrane Library, EMBASE, and Web of Science. After excluding the duplicates and irrelevant studies through reading titles and abstracts, we needed to read the full text of the remaining 49 articles to identify available data. A total of 39 articles without relevant outcome measures were excluded. Finally, we included 10 studies to conduct this systematic review and meta-analysis. Furthermore, 1,844 patients with Parkinson's disease were included. There were also 1,106 PD patients in the rotigotine group, and 738 PD patients were in the placebo group. For the basic characteristics of the included studies, see Table 1.

**Table 1:** Basic characteristics of the included studies.

**References**	**Study design**	**Age (mean** **±** **SD)**		**Sample size**		**Sex ratio (M/F)**		**Duration of treatment**	**Measuring tools**	**Hoehn and Yahr**		**Duration of PD (years)**	
		**Rotigotine TP**	**Placebo**	**Rotigotine TP**	**Placebo**	**Rotigotine TP**	**Placebo**			**Rotigotine TP**	**Placebo**	**Rotigotine TP**	**Placebo**
Chung et al. (10)	RCT	65.6 ± 8.9	64.9 ± 8.2	181	183	88/96	74/122	15	1. BDI-II; 2. Apathy Scale; 3. SHAPS	NR	NR	2.99 ± 3.26	2.51 ± 2.90
Antonini et al. (11)	RCT	68.0 ± 9.4	66.6 ± 9.8	120	207	129/95	67/58	12	1. NMSS; 2. PDQ-39	NR	NR	NR	NR
Hauser et al. (12)	RCT	68.1 ± 10.5	69.0 ± 11.7	36	40	27/14	22/18	21	1. NMSS; 2. BDI-II; 3. Apathy Scale; 4. SHAPS; 5. PDQ-8	NR	NR	4.9 ± 4.0	3.7 ± 3.7
Rascol et al. (13)	RCT	66.5 ± 11.9	65.3 ± 13.8	30	29	19/16	17/16	19	1. PDQ-8	NR	NR	5.9 ± 3.5	5.6 ± 4.7
Trenkwalder et al. (14)	RCT	64.8 ± 9.3	64.4 ± 10.6	178	89	123/68	61/35	12	1. NMSS; 2. BDI-II; 3. PDSS-2; 4. PDQ-8	NR	NR	4.6 ± 4.2	4.9 ± 4.6
Bhidayasiri et al. (15)	RCT	60.6 ± 9.5	63.5 ±1 2.5	17	17	NR	NR	12	1. PDSS-2; 2. PDQ-8	2.8 ± 0.8	2.9 ± 0.8	9.5 ± 6.0	8.3 ± 5.1
Castrioto et al. (6)	RCT	57.1 ± 6.5	60.9 ± 8.4	26	22	17/9	15/7	24	1. BDI-II; 2. Apathy Scale; 3. PDQ-39	NR	NR	26 ± 16.4	24 ± 18
Mizuno et al. (16)	RCT	64.8 ± 8.8	65.3 ± 7.9	162	81	61/103	42/42	20	1. PDSS-2	2.7 ± 0.6	2.8 ± 0.6	7.0 ± 4.9	7.0 ± 4.2
Pierantozzi et al. (17)	RCT	63.28 ± 2.98	64.04 ± 2.90	21	21	NR	NR	10	1. PDSS; 2. PSQI; 3. PSG	2.28 ± 0.25	2.23 ± 0.25	49.57 ± 3.58	51.33 ± 3.13
Poewe et al. (18)	RCT	64.3 ± 9.0	65.0 ± 10.0	204	101	132/201	71/100	24	1. PDQ-39	NR	NR	8.9 ± 4.4	8.5 ± 5.0

*PDQ-8, eight-item Parkinson's Disease Questionnaire; BDI-II, Beck Depression Inventory; NMSS, Parkinson's Disease Non-motor Symptom Assessment Scale; PDSS, Parkinson Disease Sleep Scale; PDSS-2, Modified Parkinson's Disease Sleep Scale; SHAPS, Snaith-Hamilton Pleasure Scale; PDQ-39, 39-item Parkinson's Disease Questionnaire; PSQI, Pittsburgh Sleep Quality Index; PSG, polysomnography; RCT, randomized controlled trial; NR, not reported; PD, Parkinson's disease; M/F, male/female; SD, standard deviation; Rotigotine TP, rotigotine transdermal patch*.

### Risk of Bias Assessment and Sensitivity Analysis

**Figure 2:**
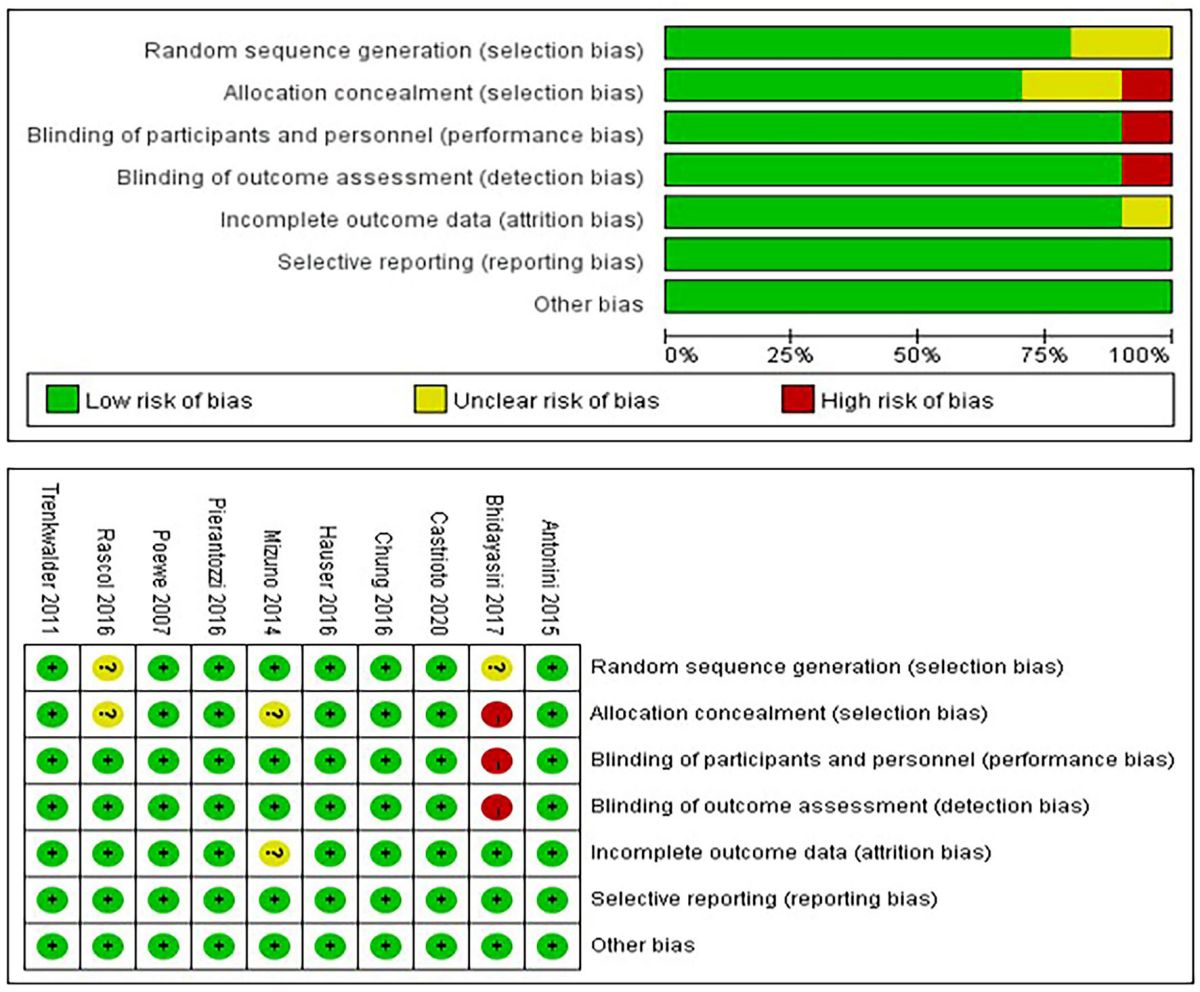
Quality assessment of the included studies.

The risk of bias was assessed by the Cochrane Risk of Bias Tool (see the results in Figure 2). In the meta-analysis of the effect of rotigotine therapy on the quality of life, we observed a significant change when the study of Castrioto et al. was deleted. The study (6) by Castrioto et al. may be a source of heterogeneity. The details are shown in Supplementary Material 2.

### Meta-Analysis Results and TSA

#### Efficacy

##### Apathy Scale

Three studies (6, 10, 12) reported the change of the Apathy Scale score from baseline to the end of treatment. The meta-analysis results showed that, compared with the placebo group, there was a significant improvement after receiving the rotigotine treatment (three studies, *n* = 488, MD = −1.68, 95% CI: −2. 74 to −0.62, *P* = 0.002; heterogeneity: χ^2^ = 0.77, *I*^2^ = 0%, *P* = 0.68). A fixed-effects model was applied.

**Figure 3:**
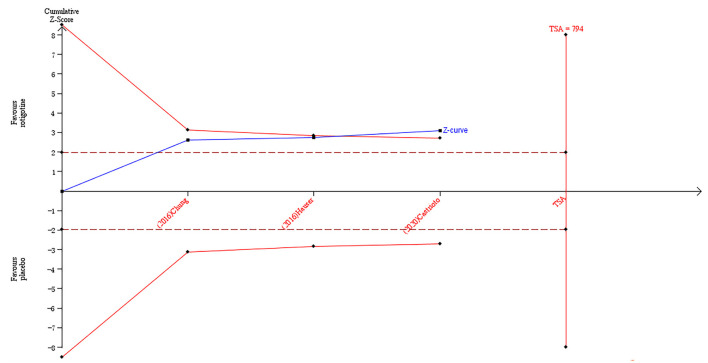
Trial sequential analysis of the cumulative meta-analysis of the effect of rotigotine vs. placebo on Apathy Scale score in Parkinson's disease patients.

The result of TSA on the data of the change of the Apathy Scale score is presented in Figure 3. The accumulated *Z*-value of meta-analysis crossed both the TSA boundary value and the traditional boundary value before the required information size of 794 was reached.

##### BDI-II

Four studies (6, 10, 12, 14) reported the change of the BDI-II score from baseline to the end of treatment. The meta-analysis results showed that, compared with the placebo group, there was a significant improvement after receiving the rotigotine treatment (four studies, *n* = 754, MD = −1.19, 95% CI: −2. 26 to −0.11, *P* = 0.03; heterogeneity: χ^2^ = 1. 60, *I*^2^ = 0%, *P* = 0.66). A fixed-effects model was applied.

**Figure 4:**
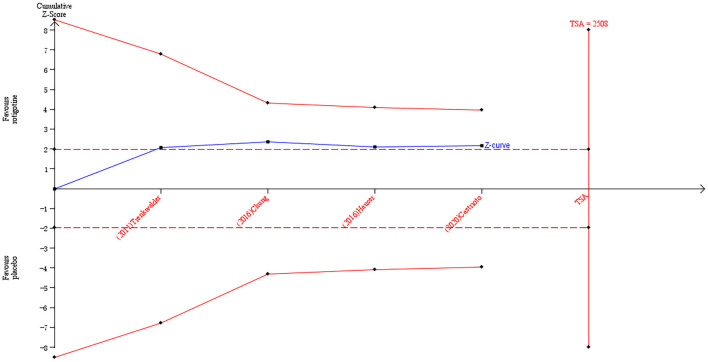
Trial sequential analysis of the cumulative meta-analysis of the effect of rotigotine vs. placebo on BDI-II score in Parkinson's disease patients.

The result of TSA on the data of the change of the BDI-II score is presented in Figure 4. The accumulated *Z*-value of meta-analysis crossed the traditional boundary value before the required information size of 2,508 was reached but did not cross the TSA boundary value.

##### NMSS

Three studies (11, 12, 14) reported the changes of NMSS total score from baseline to the end of treatment. There are nine domains in the NMSS scales, including mood/apathy, cardiovascular, perception/hallucination, attention/memory, gastrointestinal tract, urinary, sexual function, miscellaneous, and sleep/fatigue. The meta-analysis results showed that, compared with the placebo group, there was a significant improvement after receiving the rotigotine treatment (three studies, *n* = 661, MD = −3. 66, 95% CI: −4. 30 to −3. 01, *P* < 0.00001; heterogeneity: χ^2^ = 0.77, *I*^2^ = 0%, *P* = 0.68). A fixed-effects model was applied.

**Figure 5:**
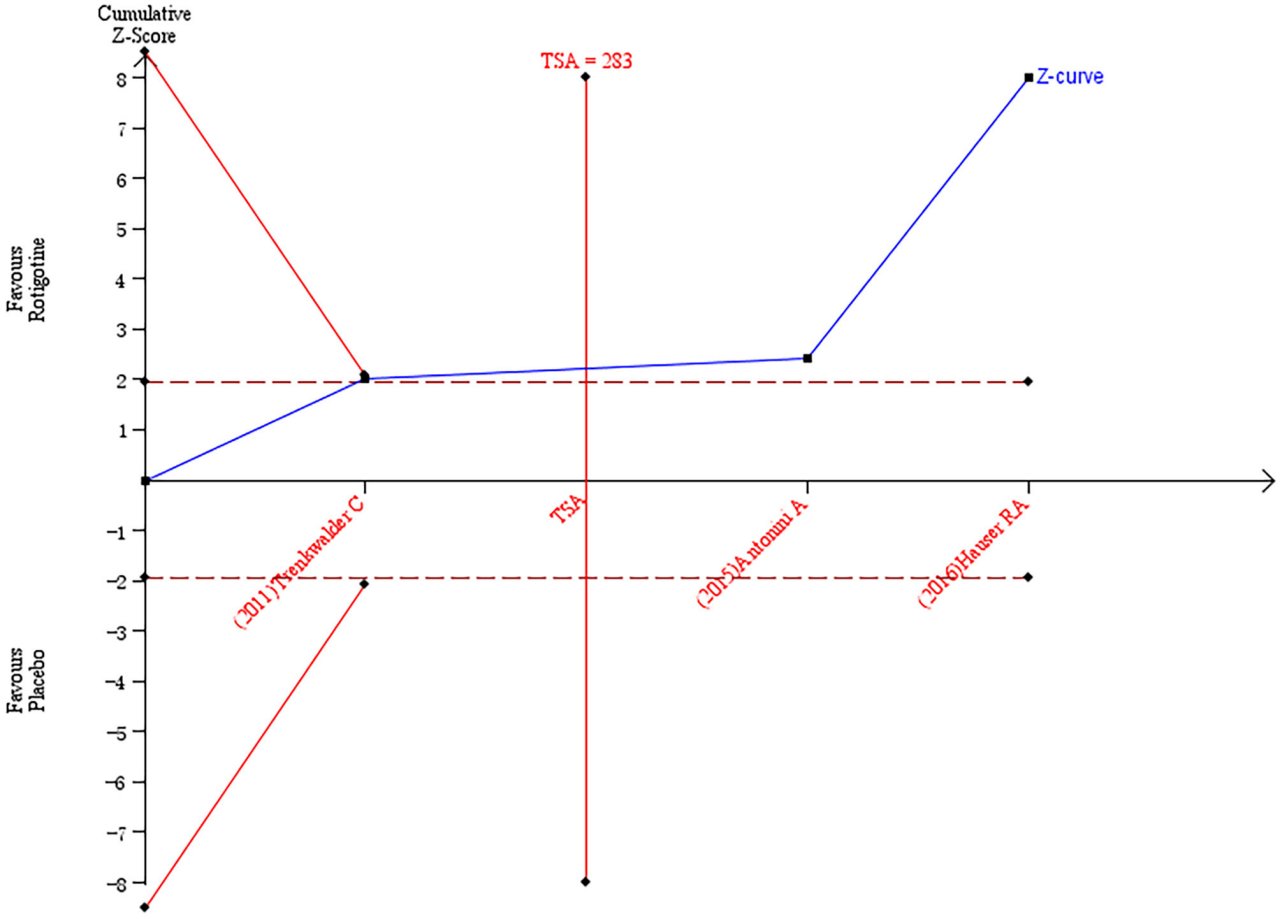
Trial sequential analysis of the cumulative meta-analysis of the effect of rotigotine vs. placebo on Parkinson's Disease Non-motor Symptom Assessment Scale total score in Parkinson's disease patients.

The result of TSA on the data of the change of NMSS total score is presented in Figure 5. The accumulated *Z*-value of meta-analysis crossed both the TSA boundary value and the traditional boundary value, and the required information size of 283 was reached.

##### Sleep/Fatigue Domain of NMSS

Three studies (11, 12, 14) reported the change of sleep/fatigue score of NMSS from baseline to the end of treatment. The meta-analysis results showed that, compared with the placebo group, there was a significant improvement after receiving rotigotine treatment (three studies, *n* = 667, MD = −2.03, 95% CI: −3. 08 to −0.98, *P* = 0.0001; heterogeneity: χ^2^ = 1. 77, *I*^2^ = 0%, *P* = 0.41). A fixed-effects model was applied.

**Figure 6:**
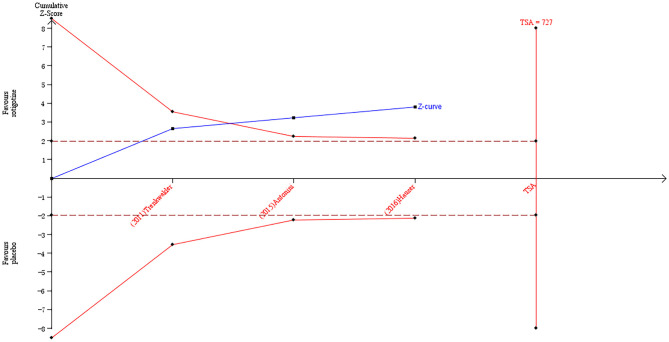
Trial sequential analysis of the cumulative meta-analysis of the effect of rotigotine vs. placebo on the sleep/fatigue score of Parkinson's Disease Non-motor Symptom Assessment Scale in Parkinson's disease patients.

The result of TSA on the data of the change of sleep/fatigue score of NMSS is presented in Figure 6. The accumulated *Z*-value of meta-analysis crossed both the TSA boundary value and the traditional boundary value before the required information size of 727 was reached.

##### Mood/Apathy Domain of NMSS

Three studies (11, 12, 14) reported the change of mood/apathy score of NMSS from baseline to the end of treatment. The meta-analysis results showed that, compared with the placebo group, there was a significant improvement after receiving the rotigotine treatment (three studies, *n* = 669, MD = −2.48, 95% CI: −4.07 to −0.89, *P* = 0.002; heterogeneity: χ^2^ = 1. 29, *I*^2^ = 0%, *P* = 0.53). A fixed-effects model was applied.

**Figure 7:**
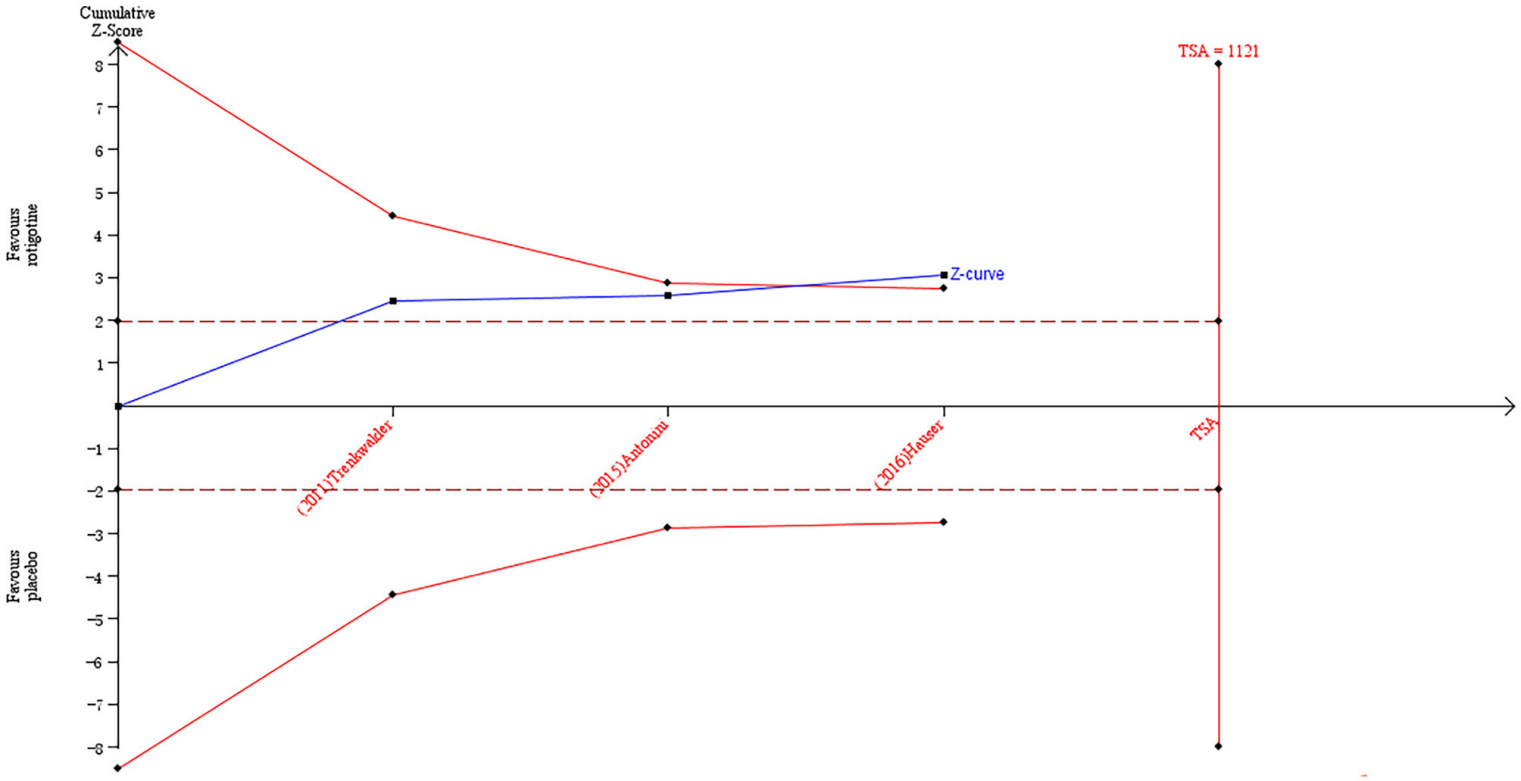
Trial sequential analysis of the cumulative meta-analysis of the effect of rotigotine vs. placebo on mood/apathy score of Parkinson's Disease Non-motor Symptom Assessment Scale in Parkinson's disease patients.

The result of TSA on the data of the change of mood/apathy score of NMSS is presented in Figure 7. The accumulated *Z*-value of meta-analysis crossed both the TSA boundary value and the traditional boundary value before the required information size of 1,121 was reached.

##### PDQ-8

Four studies (12–15) reported the change of PDQ-8 score from baseline to the end of treatment. The meta-analysis results showed that, compared with the placebo group, there was a significant improvement after receiving the rotigotine treatment (four studies, *n* = 434, MD = −4. 93, 95% CI: −6. 79 to −3. 07, *P* < 0.00001; heterogeneity: χ^2^ = 1. 70, *I*^2^ = 0%, *P* = 0.64). A fixed-effects model was applied.

**Figure 8:**
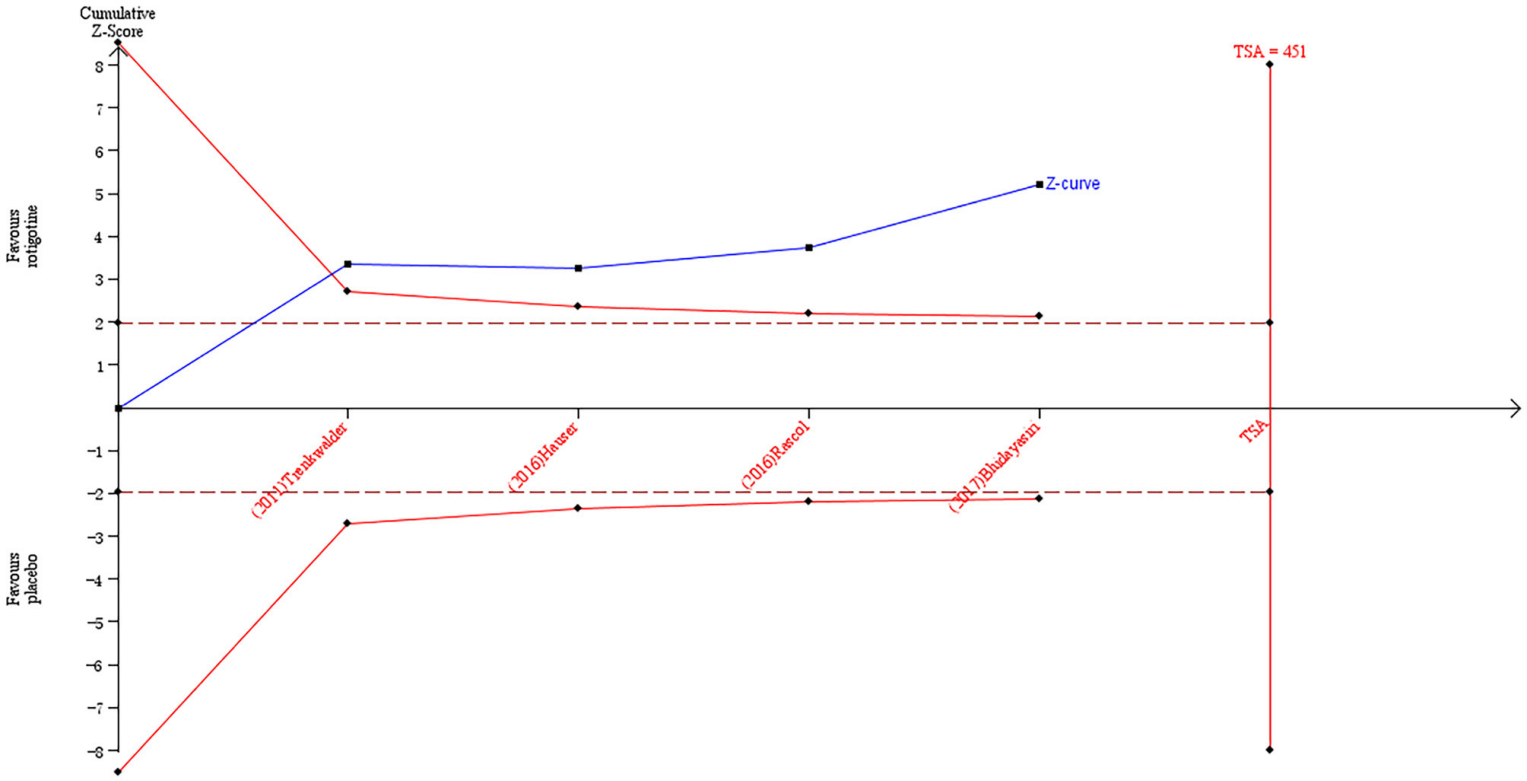
Trial sequential analysis of the cumulative meta-analysis of the effect of rotigotine vs. placebo on PDQ-8 score in Parkinson's disease patients.

The result of TSA on the data of the change of PDQ-8 score is presented in Figure 8. The accumulated *Z*-value of the meta-analysis crossed both the TSA boundary value and the traditional boundary value before the required information size of 451 was reached.

##### PDQ-39

Three studies (6, 11, 18) reported the change of PDQ-39 score from baseline to the end of treatment. The meta-analysis results showed that there was heterogeneity among the three studies. Sensitivity analysis was used to detect sources of heterogeneity. The study by Castrioto et al. was excluded based on sensitivity analysis, and the meta-analysis results showed that, compared with the placebo group, there was a significant improvement after receiving the rotigotine treatment (two studies, *n* = 620, MD = −3. 52, 95% CI: −5. 25 to −1.79, *P* < 0.0001; heterogeneity: χ^2^ = 0.01, *I*^2^ = 0%, *P* = 0.91). A fixed-effects model was applied.

**Figure 9:**
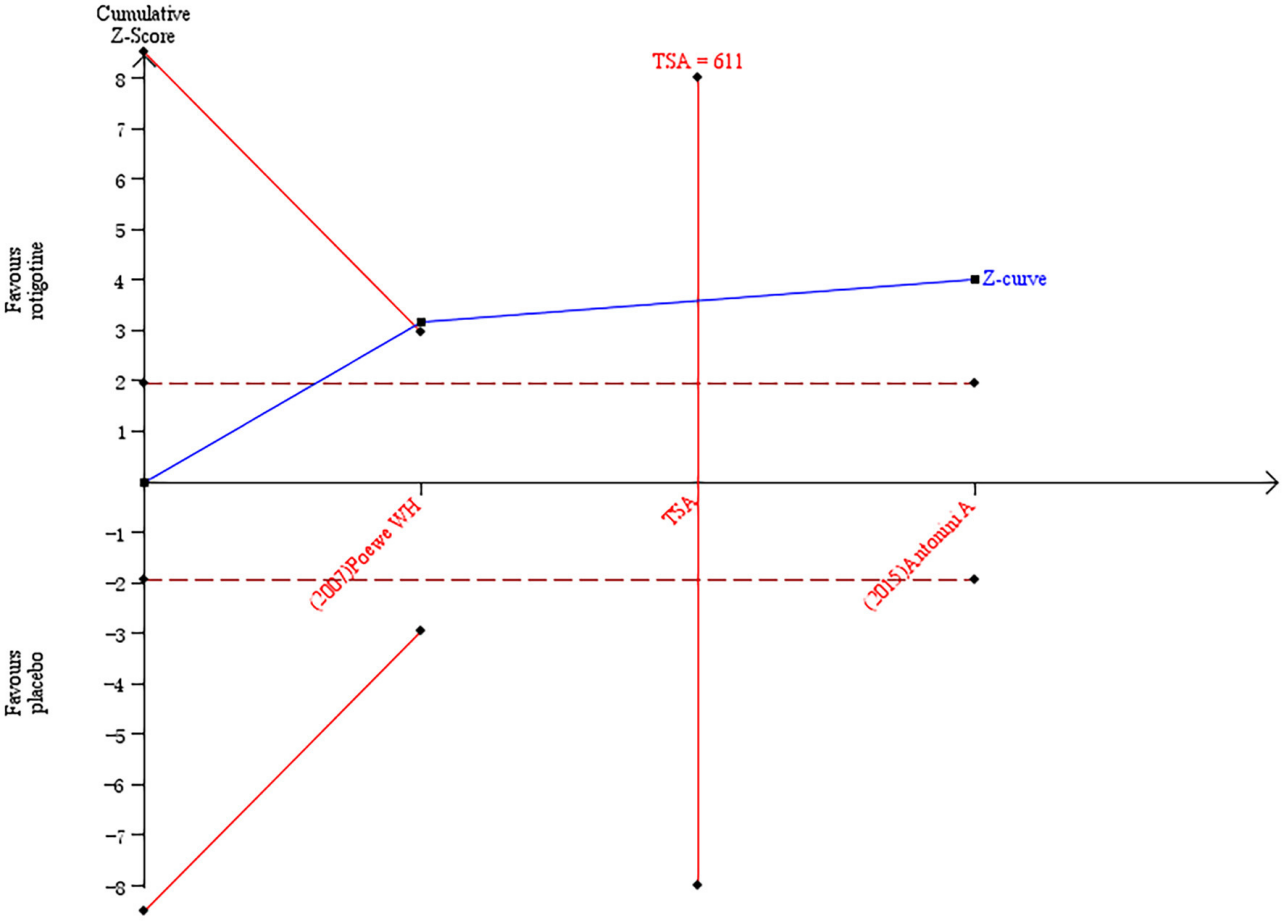
Trial sequential analysis of the cumulative meta-analysis of the effect of rotigotine vs. placebo on PDQ-39 score in Parkinson's disease patients.

The result of TSA on the data of the change of PDQ-39 score is presented in Figure 9. The accumulated *Z*-value of meta-analysis crossed both the TSA boundary value and the traditional boundary value, and the required information size of 611 was reached.

##### PDQ

Because PDQ-8 and PDQ-39 can produce equivalent results, we combined PDQ-8 and PDQ-39 using standard mean difference (SMD) to evaluate the improvement of the quality of life in PD patients. Seven studies (6, 11–15, 18) reported the change of PDQ score from baseline to the end of treatment. The meta-analysis results showed that, compared with the placebo group, there was a significant improvement after receiving the rotigotine treatment (seven studies, *n* = 1,102, SMD = −0.35, 95% CI: −0.55 to −0.15, *P* = 0.0005; heterogeneity: χ^2^ = 12.35, *I*^2^ = 51%, *P* = 0.05). Based on the sensitivity analysis, we did not detect the sources of heterogeneity. A random-effects model was applied.

##### PDSS

Three studies (14–16) reported the change of PDSS-2 score, and two studies (18, 19) reported the change of PDSS score from baseline to the end of treatment. Since the available data could not be extracted in the included studies, a qualitative analysis was performed in our study. In addition, we also recorded the effects of rotigotine on PSQI score and PSG sleep parameters. The details are shown in Table 2.

**Table 2:** Effect of rotigotine vs. placebo on sleep in patients with Parkinson's disease.

**References**	**Total cases**		**Outcome measures**	***p*-value**	**Conclusion**
	**Rotigotine**	**Placebo**			
Bhidayasiri et al. (15)	17	17	PDSS-2	*P* <0.001	Compared with placebo, the rotigotine patch provides a significant improvement in nocturnal symptoms
Mizuno et al. (16)	164	84	PDSS-2	*P* <0.001	The result showed superiority of rotigotine over placebo
Trenkwalder et al. (14)	178	89	PDSS-2	*P* <0.0001	Rotigotine treatment was associated with significant benefits vs. placebo in nocturnal sleep disturbances
Pierantozzi et al. (17)	21	21	PDSS	*P* <0.001	Compared to baseline, rotigotine treatment significantly increased the PDSS scores at the end of the study
Pierantozzi et al. (17)	21	21	PSG (SE) PSG (WASO) PSG (REM)	*P* <0.001	Rotigotine significantly increased sleep efficiency and reduced both wakefulness after sleep onset and sleep latency compared to placebo. Moreover, the mean change in REM sleep quantity was significantly higher in the rotigotine than in the placebo group
Pierantozzi et al. (17)	21	21	PSQI	*P* <0.001	Compared to baseline, the analysis of subjective sleep questionnaires of PSQI revealed that rotigotine treatment significantly reduced the PSQI global scores
Poewe et al. (18)	204	101	PDSS	*P* = 0.0129	Compared with placebo, rotigotine treatment significantly increased the PDSS scores, and rotigotine was better than placebo

##### SHAPS

Two studies (10, 12) reported the change of SHAPS score from baseline to the end of treatment. The meta-analysis results showed that there was no significant difference between two groups (two studies, *n* = 440, MD = −0.12, 95% CI: −0.58 to 0.34, *P* = 0.61; heterogeneity: χ^2^ = 0.58, *I*^2^ = 0%, *P* = 0.44). A fixed-effects model was applied.

Except for the SHAPS score, there were significant differences between the two groups. The results are shown in Table 3. The forest plots are shown in Supplementary Material 2.

**Table 3:** Effect of rotigotine vs. placebo on neuropsychiatric symptoms of Parkinson's disease.

**Outcome measures**	**Number of studies**	**Total cases**		**Statistical method**	**Heterogeneity (*I*^**2**^, %)**	**Effect size**	***P*-value**
		**Rotigotine**	**Placebo**				
Apathy Scale	3	243	245	Mean difference (IV, fixed, 95% CI)	0	−1.68 (−2.74, −0.62)	*P* = 0.002
BDI-II	4	420	334	Mean difference (IV, fixed, 95% CI)	0	−1.19 (−2.26, −0.11)	*P* = 0.03
PDQ-8	4	259	175	Mean difference (IV, fixed, 95% CI)	0	−4.93 (−6.79, −3.07)	*P* <0.0001
NMSS	3	415	246	Mean difference (IV, fixed, 95% CI)	0	−3.66 (−4.30, −3.01)	*P* <0.00001
Sleep/fatigue	3	419	248	Mean difference (IV, fixed, 95% CI)	0	−2.03 (−3.08, −0.98)	*P* = 0.0001
Mood/apathy	3	421	248	Mean difference (IV, fixed, 95% CI)	0	−2.48 (−4.07, −0.89)	*P* = 0.002
PDQ	7	689	413	Standard mean difference (IV, random, 95% CI)	51	−0.35 (−0.55, −0.15)	*P* = 0.0005
SHAPS	2	217	223	Mean difference (IV, fixed, 95% CI)	0	−0.12 (−0.58, 0.34)	*P* = 0.61
PDQ-39	2	404	216	Mean difference (IV, fixed, 95% CI)	0	−3.52 (−5.25, −1.79)	*P* <0.0001

#### Safety

##### Incidence of Nausea

Six studies (10–14, 18) reported cases of nausea from baseline to the end of treatment. The meta-analysis results showed that the incidence of nausea was higher after receiving the rotigotine treatment, and there was a significant difference between the two groups (six studies, *n* = 1,469, OR = 2. 51, 95% CI: 1. 78–3. 55, *P* < 0.00001; heterogeneity: χ^2^ = 4. 50, *P* = 0.48, *I*^2^ = 0%). A fixed-effects model was applied.

##### Incidence of Fatigue

Three studies (11–13) reported cases of fatigue from baseline to the end of treatment. The meta-analysis results showed that there was no significant difference between the two groups (three studies, *n* = 452, OR = 0.63, 95% CI: 0.27–1. 48, *P* = 0.29; heterogeneity: χ^2^ = 1. 47, *I*^2^ = 0%, *P* = 0.48). A fixed-effects model was applied.

##### Incidence of Dyskinesia

Four studies (12–14, 18) reported cases of dyskinesia from baseline to the end of treatment. The meta-analysis results showed that the incidence of dyskinesia was higher after receiving the rotigotine treatment, and there was a significant difference between the two groups (four studies, *n* = 741, OR = 2. 42, 95% CI: 1. 21–4. 82, *P* = 0.01; heterogeneity: χ^2^ = 2. 12, *I*^2^ = 0%, *P* = 0.66). A fixed-effects model was applied.

##### Incidence of Dizziness

Five studies (10, 11, 13, 14, 18) reported cases of dizziness from baseline to the end of treatment. The meta-analysis results showed that there was no significant difference between the two groups (five studies, *n* = 1,343, OR = 1. 17, 95% CI: 0.79–1. 73, *P* = 0.42; heterogeneity: χ^2^ = 3. 70, *I*^2^ = 0%, *P* = 0.45). A fixed-effects model was applied.

##### Incidence of Application and Installation Site Reactions

Seven studies (10–15, 18) reported cases of application and installation site reactions from baseline to the end of treatment. The meta-analysis results showed that the incidence of application and installation site reactions was higher after receiving the rotigotine treatment, and there was a significant difference between the two groups (seven studies, *n* = 1,458, OR = 2. 64, 95% CI: 1. 78–3. 92, *P* < 0.00001; heterogeneity: χ^2^ = 1. 68, *I*^2^ = 0%, *P* = 0.95). A fixed-effects model was applied.

##### Incidence of Insomnia

Three studies (10, 12, 13) reported cases of insomnia from baseline to the end of treatment. The meta-analysis results showed that there was heterogeneity among the included studies. We excluded the study by Hauser et al. based on a sensitivity analysis, and the meta-analysis results showed that there was no significant difference between the two groups (two studies, *n* = 448, OR = 2. 50, 95% CI: 1. 01–6. 19, *P* = 0.05; heterogeneity: χ^2^ = 0.03, *I*^2^ = 0%, *P* = 0.86). A fixed-effects model was applied.

Except for the high incidence of application and installation site reactions, nausea, and dyskinesia in the rotigotine group, there was no significant difference between the two groups. The details are shown in Table 4.

**Table 4:** Characteristic of adverse events.

**Adverse reactions**	**Number of studies**	**Event/Total (%)**		**Statistical method**	**Heterogeneity (*I*^**2**^, %)**	**Effect size**	***P*-value**
		**Rotigotine**	**Placebo**				
Application site reactions	7	129/895 (14.4)	35/563 (6.2)	Odds ratio (M-H, fixed, 95% CI)	0	2.64 (1.78, 3.92)	*P* <0.00001
Nausea	6	158/878 (18)	48/591 (8.1)	Odds ratio (M-H, fixed, 95% CI)	0	2.51 (1.78, 3.55)	*P* <0.00001
Dizziness	5	79/837 (9.4)	47/506 (9.3)	Odds ratio (M-H, fixed, 95% CI)	0	1.17 (0.79, 1.73)	*P* = 0.42
Headache	6	59/878 (6.7)	43/546 (7.9)	Odds ratio (M-H, fixed, 95% CI)	0	0.97 (0.64, 1.47)	*P* = 0.87
Insomnia	3	17/260 (6.5)	13/269 (4.8)	Odds ratio (M-H, random, 95% CI)	66	1.13 (0.19, 6.65)	*P* = 0.90
Fatigue	3	13/299 (4.3)	10/153 (6.5)	Odds ratio (M-H, fixed, 95% CI)	0	0.63 (0.27, 1.48)	*P* = 0.29
Dyskinesia	4	44/471 (9.3)	11/270 (4.1)	Odds ratio (M-H, fixed, 95% CI)	0	2.42 (1.21, 4.82)	*P* = 0.01

### Results of Rating Evidence Quality

#### Emotions

Three outcome measures, including the BDI-II score, mood/apathy score of NMSS, and AS score, were rated by the GRADE system. The evidence supporting the efficacy of rotigotine in improving the mood/apathy score of NMSS at the end of treatment was high. The evidence supporting the efficacy of rotigotine in improving the BDI-II score and the AS score at the end of treatment was moderate, and the reason for downgrading a quality rating was the inconsistency of results. The overall quality of evidence was moderate or high for the relative effects of rotigotine on emotions in patients with PD.

#### Quality of Life

Two outcome measures, including PDQ-8 and PDQ-39, were rated by the GRADE system. The evidence supporting the efficacy of rotigotine in improving the PDQ-8 score at the end of treatment was moderate, and the reason for downgrading a quality rating was that the study of Bhidayasiri did not specify the use of blind methods. The evidence supporting the efficacy of rotigotine in improving the PDQ-39 score at the end of treatment was high. The evidence for the important outcomes favored rotigotine, and the overall quality of evidence was moderate or high for the relative effects of rotigotine on the quality of life in patients with PD.

#### Sleep

One outcome measure, sleep/fatigue score of NMSS, was rated by the GRADE system. The evidence supporting the efficacy of rotigotine in improving the sleep/fatigue score of NMSS at the end of treatment was moderate, and the reason for downgrading a quality rating was the inconsistency of results. We think that the overall quality of evidence was moderate for the relative effects of rotigotine on sleep quality in patients with PD.

#### Other Non-motor Symptoms

One outcome measure, NMSS total score, was rated by the GRADE system. The evidence supporting the efficacy of rotigotine in improving the NMSS total score at the end of treatment was moderate, and the reason for downgrading a quality rating was the inconsistency of results. We think that the overall quality of evidence was moderate for the relative effects of rotigotine on non-motor symptoms in patients with PD.

Table 5 shows the GRADE evidence profile for the seven outcome measures, including the certainty of evidence.

**Table 5:** The GRADE evidence profile.

**Quality assessment**							**Number of patients**		**Effect**		**Quality**	**Importance**
**Number of studies**	**Design**	**Risk of bias**	**Inconsistency**	**Indirectness**	**Imprecision**	**Other considerations**	**Rotigotine transdermal patch**	**Placebo**	**Relative (95% CI)**	**Absolute**		
**Apathy scale (better indicated by lower values)**												
3	Randomized trials	No serious risk of bias	Serious^a^	No serious indirectness	No serious imprecision	None	243	245	-	MD 1.68 lower (2.74–0.62 lower)	⊕⊕⊕○ Moderate	Important
**PDQ-8 (better indicated by lower values)**												
4	Randomized trials	Serious^b^	No serious inconsistency	No serious indirectness	No serious	None	259	175	-	MD 4.93 lower (6.79–3.07 lower)	⊕⊕⊕○ Moderate	Important
**PDQ-39 (better indicated by lower values)**												
2	Randomized trials	No serious risk of bias	No serious inconsistency	No serious indirectness	No serious imprecision	None	404	216	-	MD 3.52 lower (5.25–1.79 lower)	⊕⊕⊕⊕ High	Important
**NMSS (better indicated by lower values)**												
3	Randomized trials	No serious risk of bias	Serious^a^	No serious indirectness	No serious imprecision	None	415	246	-	MD 3.66 lower (4.3–3.01 lower)	⊕⊕⊕○ Moderate	Important
**Fatigue/sleep (better indicated by lower values)**												
3	Randomized trials	No serious risk of bias	Serious^a^	No serious indirectness	No serious imprecision	None	419	248	-	MD 2.03 lower (3.08–0.98 lower)	⊕⊕⊕○ Moderate	Important
**Mood/apathy (better indicated by lower values)**												
3	Randomized trials	No serious risk of bias	No serious inconsistency	No serious indirectness	No serious imprecision	None	421	248	-	MD 2.48 lower (4.07–0.89 lower)	⊕⊕⊕⊕ High	Important
**BDI-II (better indicated by lower values)**												
4	Randomized trials	No serious risk of bias	Serious^a^	No serious indirectness	No serious imprecision	None	420	334	-	MD 1.19 lower (2.26–0.11 lower)	⊕⊕⊕○ Moderate	Important

*GRADE Working Group grades of evidence: high quality—further research is very unlikely to change our confidence in the estimate of effect, moderate quality—further research is likely to have an important impact on our confidence in the estimate of effect and may change the estimate, low quality—further research is very likely to have an important impact on our confidence in the estimate of effect and is likely to change the estimate, and very low quality—we are very uncertain about the estimate*.

a *The results were inconsistent among studies*.

b *The study of Bhidayasiri did not specify the use of blind methods*.

## Discussion

### Efficacy and Safety

Our results demonstrated that rotigotine played an important role in neuropsychiatric symptoms, sleep, and quality of life, which are consistent with the previous meta-analysis conducted in 2018 (5). However, the latest research was not included. Castrioto et al. (6) conducted a randomized, controlled, double-blind study of rotigotine on neuropsychiatric symptoms in 2020, and PD patients untreated by L-dopa or dopamine agonists were included. We found that it was the first randomized, controlled study specifically designed to explore the efficacy of rotigotine on neuropsychiatric symptoms in *de novo* PD, which might be contributive in exploring the real efficacy of rotigotine on neuropsychiatric symptoms without the confounding effect of antiparkinsonian drugs, but the results showed that, compared with placebo, rotigotine could significantly improve trait anxiety symptoms, but not apathy and depression. One reason why the effect was not significant in their study may be related to the use of low doses of rotigotine. They used a recommended dose in the early stages of PD (up to 8 mg/day), which was much lower than the equivalent ones used in the successful study. It has been proven that, at maintenance dosages of ≤ 8 mg/24 h, monotherapy with the rotigotine can significantly improve the motor symptoms of patients with early Parkinson's disease (20–22), but randomized, controlled studies which explored the efficacy of rotigotine on non-motor symptoms in *de novo* PD were scarce, so further research will be needed to explore the efficacy.

Up to 96% of Parkinson's disease patients suffer from sleep disorders, but there is no formal instrument to quantify all aspects of nocturnal sleep problems in Parkinson's disease (23–25). Subjective scales were frequently used in the included studies, including PSQI, PDSS, PDSS-2, and the sleep/fatigue subdomain of the NMSS. The effect of rotigotine on reducing subjective sleep disturbances has been confirmed, and our results are consistent with previous studies (3, 14, 26), but only one study used objective instruments to assess the effects of rotigotine on sleep structure. We found that the study (17) by Pierantozzi et al. was the first randomized controlled trial that used PSG recordings to objectively investigate the effect of rotigotine on the sleep architecture of PD and got a positive conclusion that rotigotine, which was limited to nocturnal administration, might improve sleep quality and continuity in patients with PD. PSG has become an effective method to study sleep structure, but studies which investigated the effect of rotigotine on the sleep architecture of PD using PSG recordings were rare, so further studies will be still needed.

Rotigotine can effectively improve neuropsychiatric symptoms, quality of life, and sleep in PD, but at the same time, it also brings some side effects. In our study, we found that, compared with placebo, there were significant differences in application site reactions, nausea, and dyskinesia, but most of the reactions were mild or moderate. Similar to dopamine receptor agonists, there were some adverse events after receiving the rotigotine treatment, such as dizziness, headache, insomnia, or fatigue. However, there was no significant difference.

Rotigotine is a non-ergolinic D3/D2 and D1 dopamine agonist. Impulse control disorders which are mainly associated with dopamine D2/D3 agonists are frequent side effects of dopamine replacement treatment used in PD patients (27, 28). It was reported that impulse control disorders occur in about 17% of PD patients on dopamine agonists, which include binge eating disorder, compulsive sexual behavior, gambling disorder, and compulsive shopping (27). However, only one article reported the occurrence of impulse control disorders in our included literature. In the study of Trenkwalder et al. (14), the authors used the modified Minnesota Impulsive Disorder Interview (mMIDI) to monitor the emergence of impulse control disorders. The results showed that nine subjects had a positive result on at least one mMIDI module [placebo, 2 (2%); rotigotine, 7 (4%)]. On the structured psychiatric interview, the authors found a rotigotine-treated subject who had positive findings of compulsive sexual behavior, but it was not reported as an impulse control disorder.

Different dopamine receptor agonists have different effects on the D3 receptor. A study showed that taking pramipexole or ropinirole led to a higher risk of impulse control disorder, and there was a dose–response relationship (28). In the study of Trenkwalder et al. (14), the incidence of impulse control disorders after receiving rotigotine treatment (4%) was much lower than the incidence of other dopamine receptor agonists (17%). It has been proven in our meta-analysis that rotigotine has no serious adverse reactions and has good safety and tolerability.

### Analysis of the TSA Results

According to the results of TSA, conclusive evidence supporting the efficacy of rotigotine in improving the AS score, NMSS total score, sleep/fatigue score of NMSS, mood/apathy score of NMSS, PDQ-8 score, PDQ-39 score, and PDQ score have been obtained in the rotigotine group, but the result of TSA on the data of the change of the BDI-II score implied that conclusive evidence has not been obtained in the rotigotine group compared with the placebo group. This means that the traditional meta-analysis may have a false-positive conclusion and means that more trials need to be included to confirm the efficacy.

### Analysis of GRADE Results

Seven outcome measures of clinical efficacy were objectively rated by the GRADE system. Evidence supporting the efficacy of rotigotine in improving the BDI-II score, AS score, PDQ-8 score, sleep/fatigue score of NMSS, and NMSS total score were moderate, and evidence in improving the mood/apathy score of NMSS and PDQ-39 were high. We found that the main reasons for downgrading a quality rating are the inconsistency of results among studies and risk of bias. In future research, we should avoid the abovementioned shortcomings. Rotigotine is a non-ergolinic dopamine agonist. It is administered once a day *via* a transdermal patch, and its effect lasts for 24 h (29, 30). It has been approved for the treatment of PD in the EU and other numerous countries, such as China, Australia, USA, and Japan (31). The overall evidence of rotigotine in the treatment of neuropsychiatric symptoms in PD patients is good, and it is worthy of clinical application.

### Strengths and Limitations

There are several major strengths in our study compared with the previous meta-analysis conducted in 2018 (5): (1) The first strength of this study includes the systematic data collection methods and study design, being both a systematic review and a meta-analysis, so the results can be fully displayed; (2) More studies were included to conduct this meta-analysis; (3) In our study, TSA is used to test whether the conclusions of the meta-analysis are sufficient; and (4) The quality of evidence for each outcome was rated by the GRADE system.

Our study has several potential limitations: (1) Most patients in the included studies used rotigotine or placebo based on the use of dopamine preparations or dopamine receptor agonists. There was only one randomized controlled trial specifically designed to assess the efficacy of rotigotine on neuropsychiatric symptoms in *de novo* PD, without the confounding effect of antiparkinsonian drugs; (2) The dosage of rotigotine used in each included study was different, and the duration of each study was different; (3) The severity of Parkinson's disease varies; the final scale score may be affected by baseline differences among participants; and (4) The required information size was estimated based on a type I error of 5%, a type II error of 20%, and the expected mean difference and variation which was estimated by combined meta-analysis in the included trials with a low risk of bias. This may result in an overestimation of the sample size.

## Conclusions

Our systematic review and meta-analysis demonstrate that rotigotine can effectively improve neuropsychiatric symptoms and exerts a positive effect on sleep and quality of life in Parkinson's disease, with good safety and tolerability.

## Data Availability Statement

The original contributions presented in the study are included in the article/Supplementary Material, further inquiries can be directed to the corresponding authors.

## Author Contributions

JYan and JYang designed the study. HM, AL, and JH carried out literature searches, study selection, and data extraction. HM and AL assessed the quality of studies, rated the quality of evidence, conducted trial sequential analysis, contributed to the analysis, and interpreted the data. HM wrote the manuscript. JYan revised the manuscript. All authors contributed to the writing of this manuscript.

## Funding

This work was supported by the Project of Henan Province Science and Technology (202102310216), the Key Projects of Medical Science and Technology in Henan Province (SBGJ202002099), and the Medical Science and Technology Research in Henan Province (LHGJ20190560).

## Conflict of Interest

The authors declare that the research was conducted in the absence of any commercial or financial relationships that could be construed as a potential conflict of interest.

## Publisher's Note

All claims expressed in this article are solely those of the authors and do not necessarily represent those of their affiliated organizations, or those of the publisher, the editors and the reviewers. Any product that may be evaluated in this article, or claim that may be made by its manufacturer, is not guaranteed or endorsed by the publisher.

## References

[1] Barone P Antonini A Colosimo C Marconi R Morgante L Avarello TP . The PRIAMO study: a multicenter assessment of nonmotor symptoms and their impact on quality of life in Parkinson's disease. Mov Disord. (2009) 24:1641–9. 10.1002/mds.22643 19514014

[2] Poewe W. Non-motor symptoms in Parkinson's disease. Eur J Neurol. (2008) 15(Suppl. 1):14–20. 10.1111/j.1468-1331.2008.02056.x 18353132

[3] Jenner P. A novel dopamine agonist for the transdermal treatment of Parkinson's disease. Neurology. (2005) 65(Suppl. 1):S3–5. 10.1212/WNL.65.2_suppl_1.S3 16030291

[4] Pfeiffer RF. A promising new technology for Parkinson's disease. Neurology. (2005) 65(Suppl. 1):S6–10. 10.1212/WNL.65.2_suppl_1.S6 16030292

[5] Wang HT Wang L He Y Yu G. Rotigotine transdermal patch for the treatment of neuropsychiatric symptoms in Parkinson's disease: a meta-analysis of randomized placebo-controlled trials. J Neurol Sci. (2018) 393:31–8. 10.1016/j.jns.2018.08.003 30099246

[6] Castrioto A Thobois S Anheim M Quesada JL Lhommée E Klinger H . A randomized controlled double-blind study of rotigotine on neuropsychiatric symptoms in *de novo* PD. NPJ Parkinsons Dis. (2020) 6:41. 10.1038/s41531-020-00142-x 33319786 PMC7738499

[7] Wetterslev J Jakobsen JC Gluud C. Trial sequential analysis in systematic reviews with meta-analysis. BMC Med Res Methodol. (2017) 17:39. 10.1186/s12874-017-0315-7 28264661 PMC5397700

[8] Wetterslev J Thorlund K Brok J Gluud C. Trial sequential analysis may establish when firm evidence is reached in cumulative meta-analysis. J Clin Epidemiol. (2008) 61:64–75. 10.1016/j.jclinepi.2007.03.013 18083463

[9] Guyatt G Oxman AD Akl EA Kunz R Vist G Brozek J . Schunemann: GRADE guidelines: 1. Introduction-GRADE evidence profiles and summary of findings tables. J Clin Epidemiol. (2011) 64:383–94. 10.1016/j.jclinepi.2010.04.026 21195583

[10] Chung SJ Asgharnejad M Bauer L Ramirez F Jeon B. Evaluation of rotigotine transdermal patch for the treatment of depressive symptoms in patients with Parkinson's disease. Expert Opin Pharmacother. (2016) 17:1453–61. 10.1080/14656566.2016.1202917 27322571

[11] Antonini A Bauer L Dohin E Oertel WH Rascol O Reichmann H . Effects of rotigotine transdermal patch in patients with Parkinson's disease presenting with non-motor symptoms - results of a double-blind, randomized, placebo-controlled trial. Eur J Neurol. (2015) 22:1400–7. 10.1111/ene.12757 26095948

[12] Hauser RA Slawek J Barone P Dohin E Surmann E Asgharnejad M . Evaluation of rotigotine transdermal patch for the treatment of apathy and motor symptoms in Parkinson's disease. BMC Neurol. (2016) 16:90. 10.1186/s12883-016-0610-7 27267880 PMC4895976

[13] Rascol O Zesiewicz T Chaudhuri KR Asgharnejad M Surmann E Dohin E . A randomized controlled exploratory pilot study to evaluate the effect of rotigotine transdermal patch on Parkinson's disease-associated chronic pain. J Clin Pharmacol. (2016) 56:852–61. 10.1002/jcph.678 26626320

[14] Trenkwalder C Kies B Rudzinska M Fine J Nikl J Honczarenko K . Chaudhuri: Rotigotine effects on early morning motor function and sleep in Parkinson's disease: a double-blind, randomized, placebo-controlled study (RECOVER). Mov Disord. (2011) 26:90–9. 10.1002/mds.23441 21322021 PMC3072524

[15] Bhidayasiri R Sringean J Chaiwong S Anan C Penkeaw N Leaknok A . Rotigotine for nocturnal hypokinesia in Parkinson's disease: Quantitative analysis of efficacy from a randomized, placebo-controlled trial using an axial inertial sensor. Parkinsonism Relat Disord. (2017) 44:124–8. 10.1016/j.parkreldis.2017.08.010 28818560

[16] Mizuno Y Nomoto M Hasegawa K Hattori N Kondo T Murata M . Rotigotine trial: rotigotine vs. ropinirole in advanced stage Parkinson's disease: a double-blind study. Parkinsonism Relat Disord. (2014) 20:1388–93. 10.1016/j.parkreldis.2014.10.005 25455692

[17] Pierantozzi M Placidi F Liguori C Albanese M Imbriani P Marciani MG . Stefani: Rotigotine may improve sleep architecture in Parkinson's disease: a double-blind, randomized, placebo-controlled polysomnographic study. Sleep Med. (2016) 21:140–4. 10.1016/j.sleep.2016.01.016 27448485

[18] Poewe WH Rascol O Quinn N Tolosa E Oertel WH Martignoni E . Efficacy of pramipexole and transdermal rotigotine in advanced Parkinson's disease: a double-blind, double-dummy, randomised controlled trial. Lancet Neurol. (2007) 6:513–20. 10.1016/S1474-4422(07)70108-4 17509486

[19] Fei L Zhou D Ding TZ. The efficacy and safety of rotigotine transdermal patch for the treatment of sleep disorders in Parkinson's disease: a meta-analysis. Sleep Med. (2019) 61:19–25. 10.1016/j.sleep.2019.05.002 31272824

[20] Jankovic J Watts RL Martin W Boroojerdi B. Transdermal rotigotine: double-blind, placebo-controlled trial in Parkinson disease. Arch Neurol. (2007) 64:676–82. 10.1001/archneur.64.5.676 17502466

[21] Watts RL Jankovic J Waters C Rajput A Boroojerdi B Rao J. Randomized, blind, controlled trial of transdermal rotigotine in early Parkinson disease. Neurology. (2007) 68:272–6. 10.1212/01.wnl.0000252355.79284.22 17202432

[22] Zhang ZX Shang HF Hu X Chen S Zhao Z Du X . Rotigotine transdermal patch in Chinese patients with early Parkinson's disease: a randomized, double-blind, placebo-controlled pivotal study. Parkinsonism Relat Disord. (2016) 28:49–55. 10.1016/j.parkreldis.2016.04.022 27172830

[23] Chaudhuri KR Healy DG Schapira HA. Non-motor symptoms of Parkinson's disease: diagnosis and management. Lancet Neurol. (2006) 5:235–45. 10.1016/S1474-4422(06)70373-8 16488379

[24] Chaudhuri KR Pal S DiMarco A Whately-Smith C Bridgman K Mathew R . The Parkinson's disease sleep scale: a new instrument for assessing sleep and nocturnal disability in Parkinson's disease. J Neurol Neurosurg Psychiatry. (2002) 73:629–35. 10.1136/jnnp.73.6.629 12438461 PMC1757333

[25] Poewe W Högl B. Parkinson's disease and sleep. Curr Opin Neurol. (2000) 13:423–6. 10.1097/00019052-200008000-00009 10970059

[26] Giladi N Fichtner A Poewe W Boroojerdi B. Rotigotine transdermal system for control of early morning motor impairment and sleep disturbances in patients with Parkinson's disease. J Neural Transm (Vienna). (2010) 117:1395–9. 10.1007/s00702-010-0506-4 21080009

[27] Voon V Napier TC Frank MJ Sgambato-Faure V Grace AA Rodriguez-Oroz M . Impulse control disorders and levodopa-induced dyskinesias in Parkinson's disease: an update. Lancet Neurol. (2017) 16:238–50. 10.1016/S1474-4422(17)30004-2 28229895

[28] Corvol JC Artaud F Cormier-Dequaire F Rascol O Durif F Derkinderen P . Group: Longitudinal analysis of impulse control disorders in Parkinson disease. Neurology. (2018) 91:e189–201. 10.1212/WNL.0000000000005816 29925549 PMC6059034

[29] Baldwin CM Keating MG. Rotigotine transdermal patch: a review of its use in the management of Parkinson's disease. CNS Drugs. (2007) 21:1039–55. 10.2165/00023210-200721120-00007 18020483

[30] Sanford M Scott JL. Rotigotine transdermal patch: a review of its use in the treatment of Parkinson's disease. CNS Drugs. (2011) 25:699–719. 10.2165/11206750-000000000-00000 21790211

[31] Frampton JE. Rotigotine transdermal patch: a review in Parkinson's disease. CNS Drugs. (2019) 33:707–18. 10.1007/s40263-019-00646-y 31243728

