# Supplementary material for: Efficacy and Safety of Rotigotine Transdermal Patch on Neuropsychiatric Symptoms of Parkinson's Disease: An Updated Meta-Analysis and Systematic Review
Source: Front Neurol. 2021 Oct 21;12:722892. doi: 10.3389/fneur.2021.722892 (PMC8567083; doi:10.3389/fneur.2021.722892)

**Supplementary Material 1 :** **The Search Strategy on PubMed**

- **#1 (**Parkinson Disease [mh] ) OR Idiopathic Parkinson's Disease OR Lewy Body Parkinson's Disease OR Parkinson's Disease, Idiopathic OR Parkinson's Disease, Lewy Body OR Parkinson Disease, Idiopathic OR Parkinson's Disease OR Idiopathic Parkinson Disease OR Lewy Body Parkinson Disease OR Primary Parkinsonism OR Parkinsonism, Primary OR PD OR Paralysis Agitans
- #2 (rotigotine [mh] ) OR 2-(N-n-propyl-N-2-thienylethylamino)-5-hydroxytetralin OR N 0437, hydrochloride, (S)-isomer OR N 0923 OR N-0923 OR N 0924 OR N-0924 OR rotigotine, (+)- OR (+)-5,6,7,8-tetrahydro-6-(propyl(2-(2-thienyl)ethyl)amino)-1-naphthol OR Neupro OR N 0437 OR N-0437 OR N 0437, (+-)-isomer OR N 0437, (-)-isomer OR N 0437, hydrochloride, (R)-isomer OR rotigotine, (+--)- OR racemic N-0437 OR rotigotine (+-)-form OR 1-naphthalenol,5,6,7,8-tetrahydro-6-(propyl(2-(2-thienyl)ethyl)amino)- OR (+--)-5,6,7,8-tetrahydro-6-(propyl(2-(2-thienyl)ethyl)amino)-1-naphthol OR N 0437, (R)-isomer OR Rotigotine CDS
- #3 **#1** AND #2
- #4 randomized controlled trial [pt]
- #5 controlled clinical trial [pt]
- #6 randomized [ti/ab]
- #7 placeb [ti/ab]
- #8 randomly [ti/ab]
- #9 tria [ti/ab]
- #10 groups [ti/ab]
- #11 #4 OR #5 OR #6 OR #7 OR #8 OR #9 OR #10
- #12 #3 AND #11

**Supplementary Material 2 : The Forest plot and sensitivity analysis.**

Forest plot of the change of the Apathy Scale score at the end of treatment.


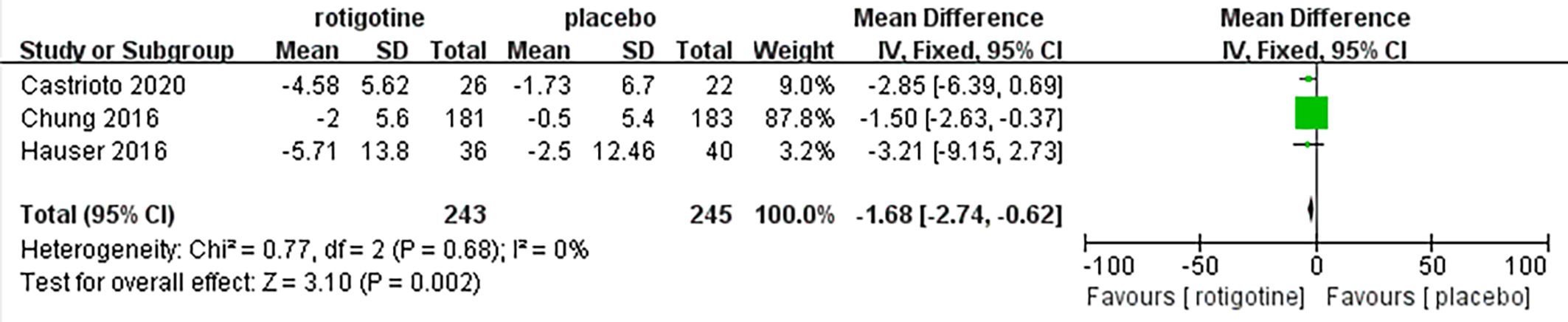


Forest plot of the change of the BDI-II score at the end of treatment.


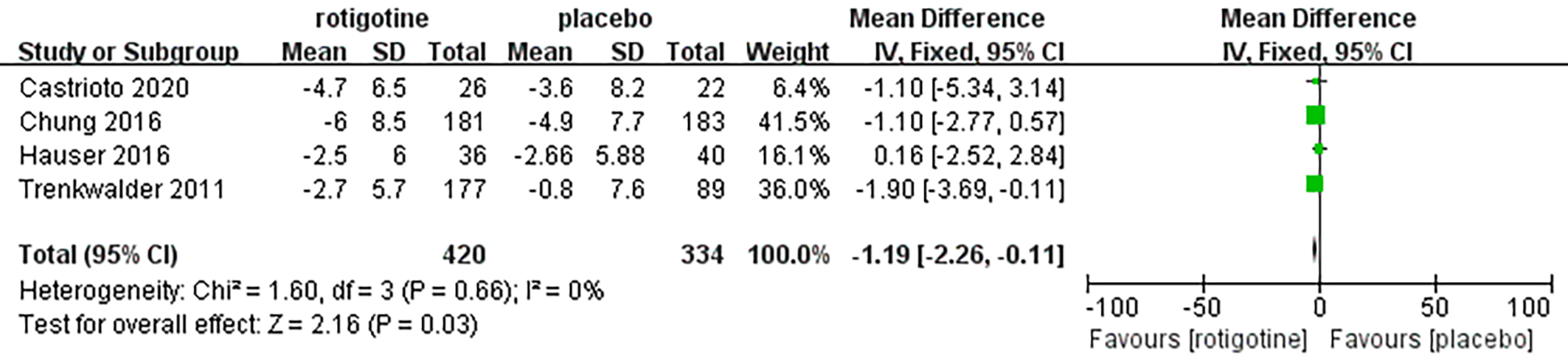


Forest plot of the change of NMSS total score at the end of treatment.


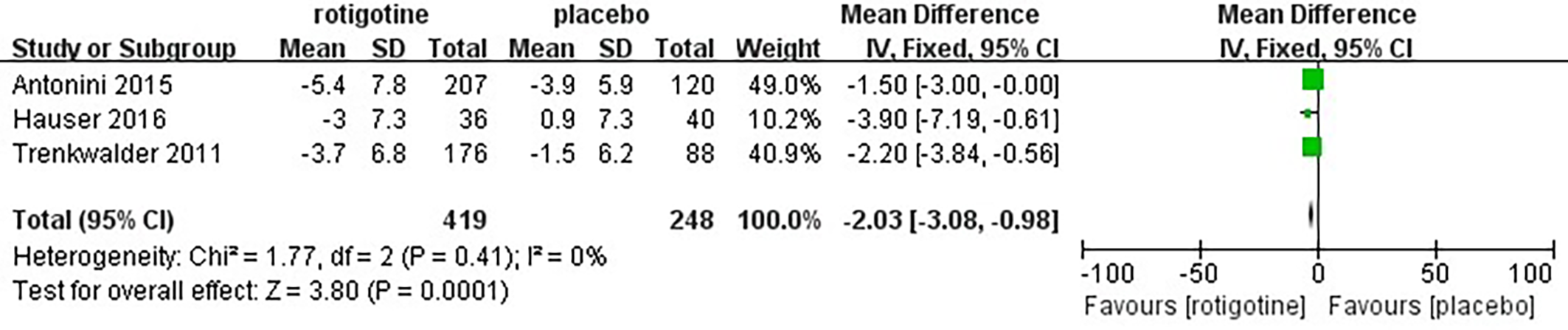


Forest plot of the change of sleep/fatigue score of NMSS at the end of treatment.


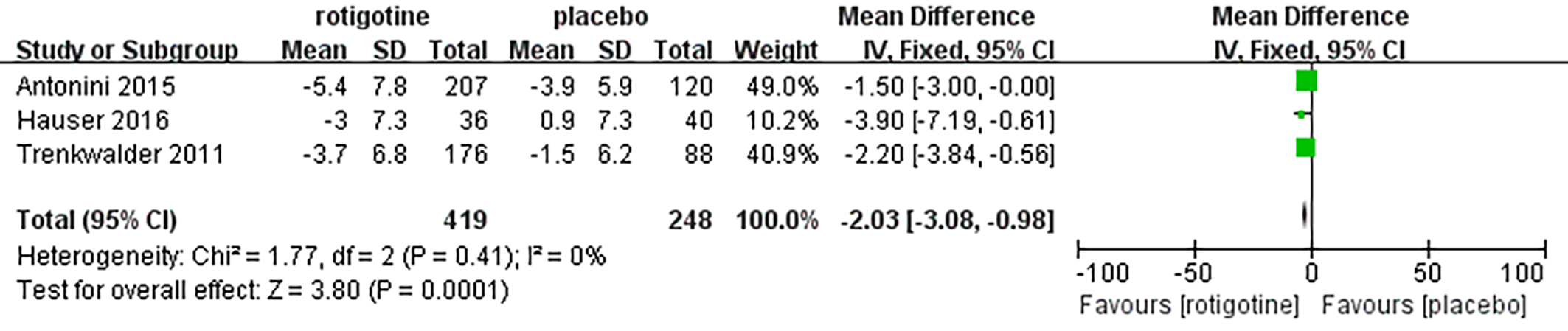


Forest plot of the change of mood/apathy score of NMSS at the end of treatment.


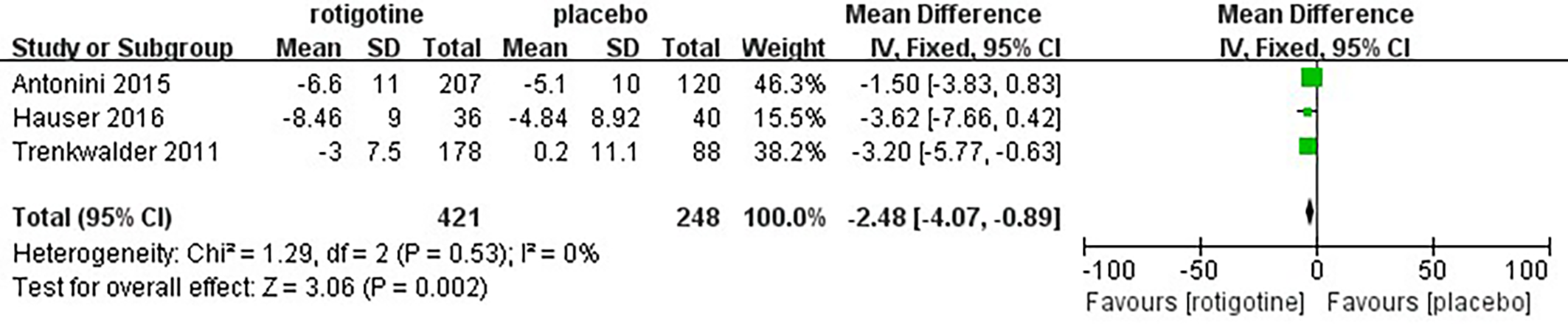


Forest plot of the change of PDQ-8 score at the end of treatment.


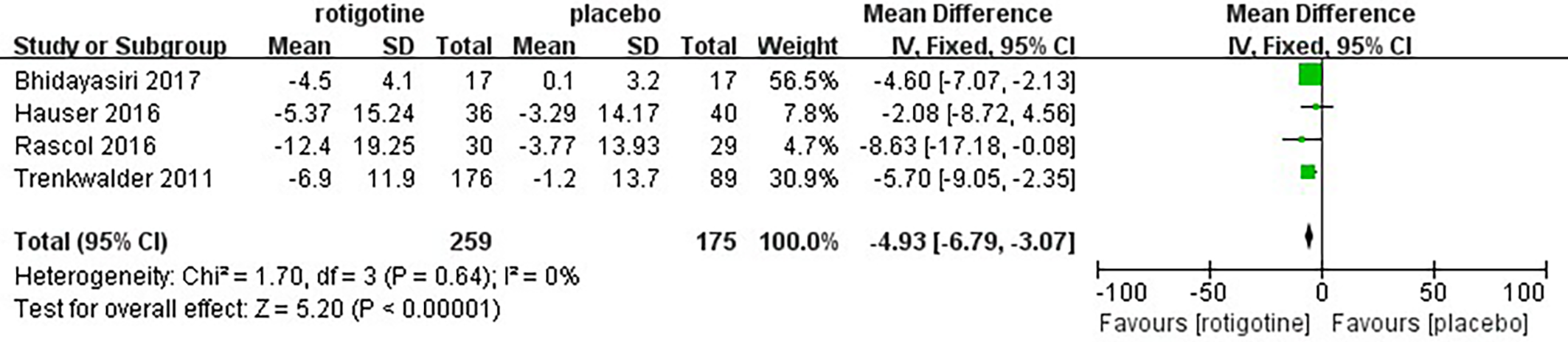


Forest plot of the change of PDQ-39 score at the end of treatment.


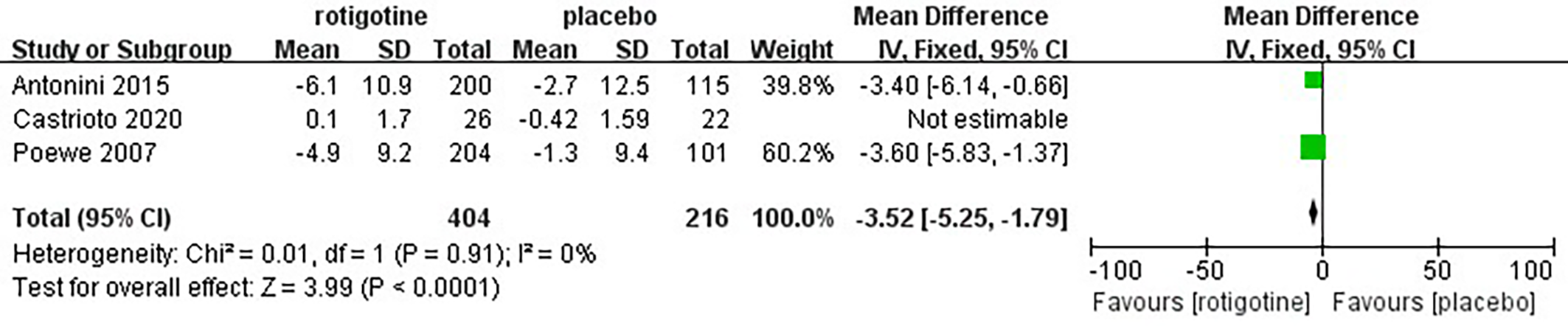


Forest plot of PDQ score.


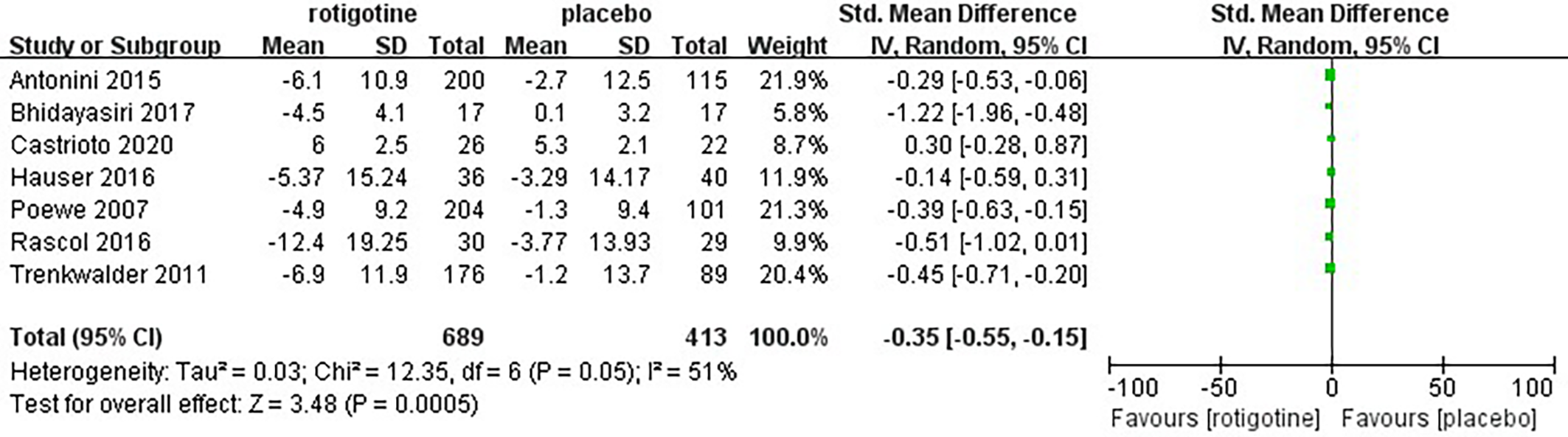


Forest plot of the change of SHAPS score at the end of treatment.


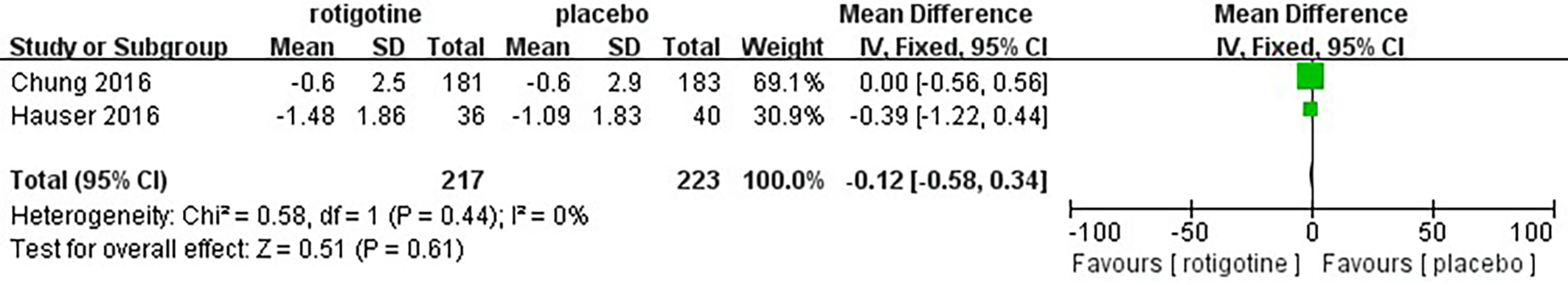


Forest plot of incidence of nausea.


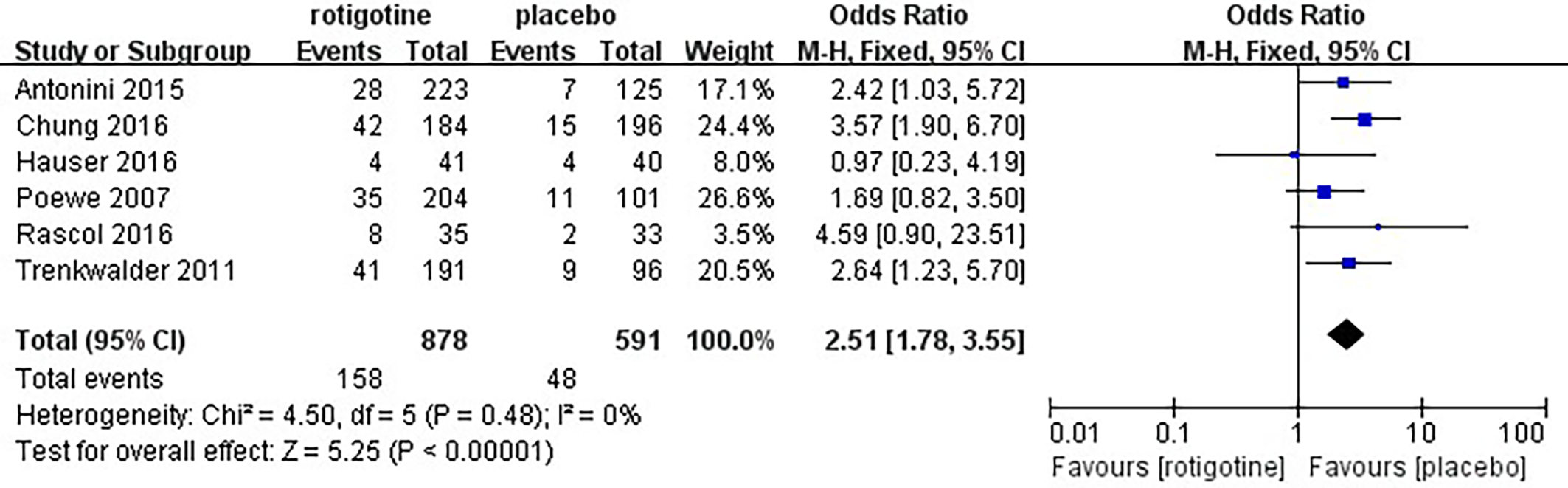


Forest plot of incidence of fatigue.


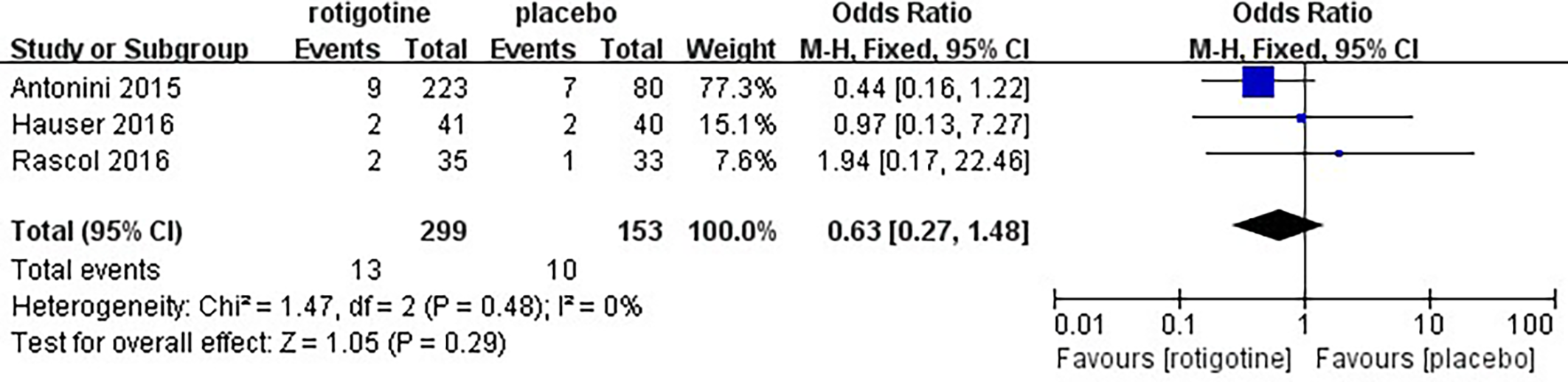


Forest plot of incidence of dyskinesia.


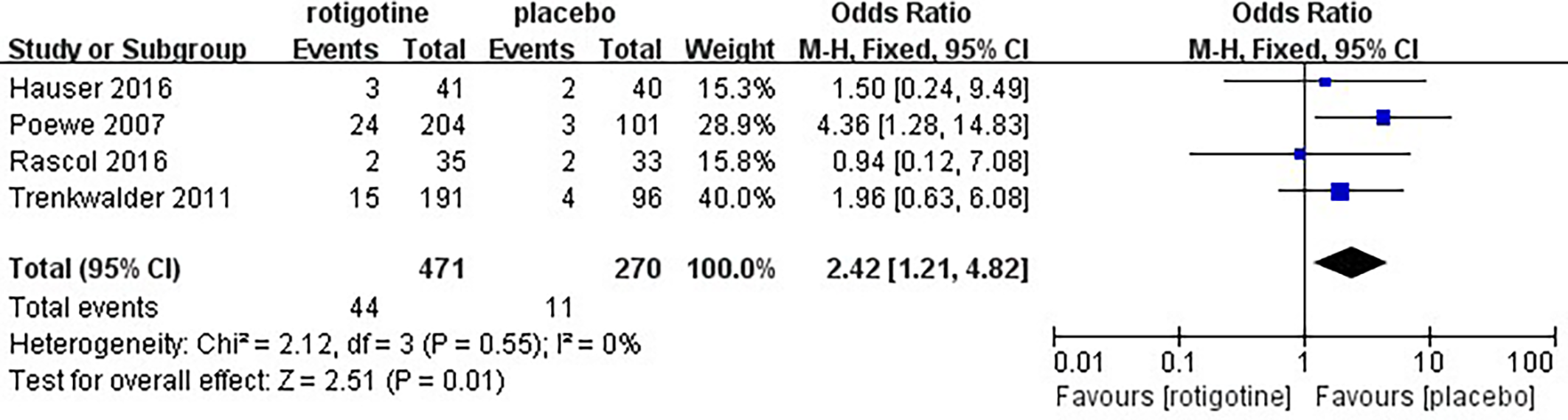


Forest plot of incidence of dizziness.


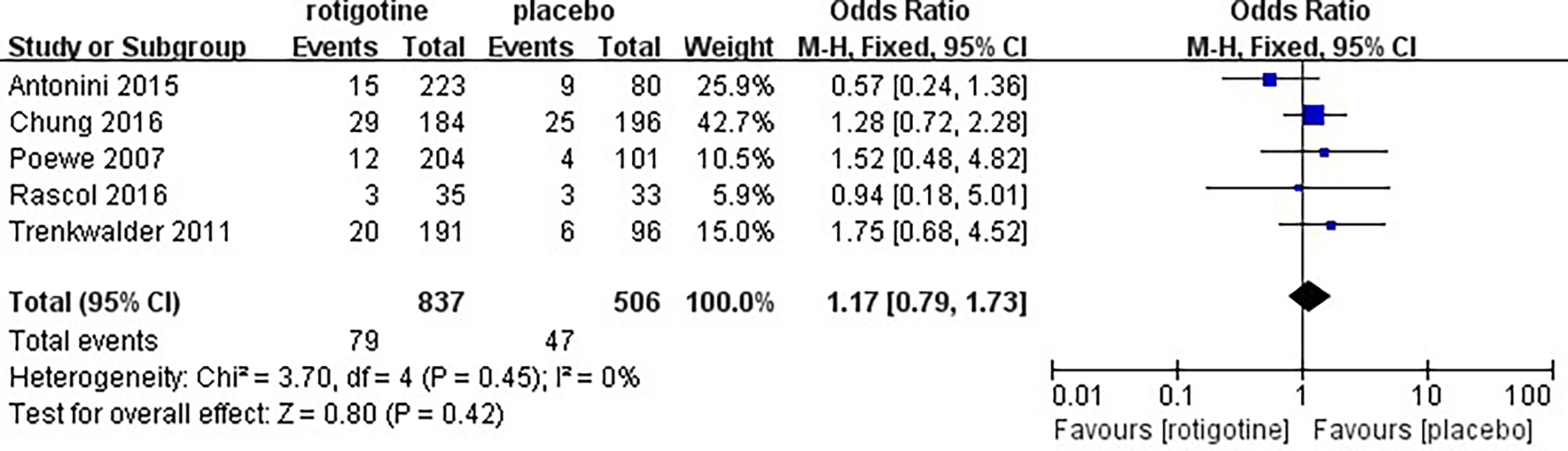


Forest plot of incidence of application and instillation site reactions.


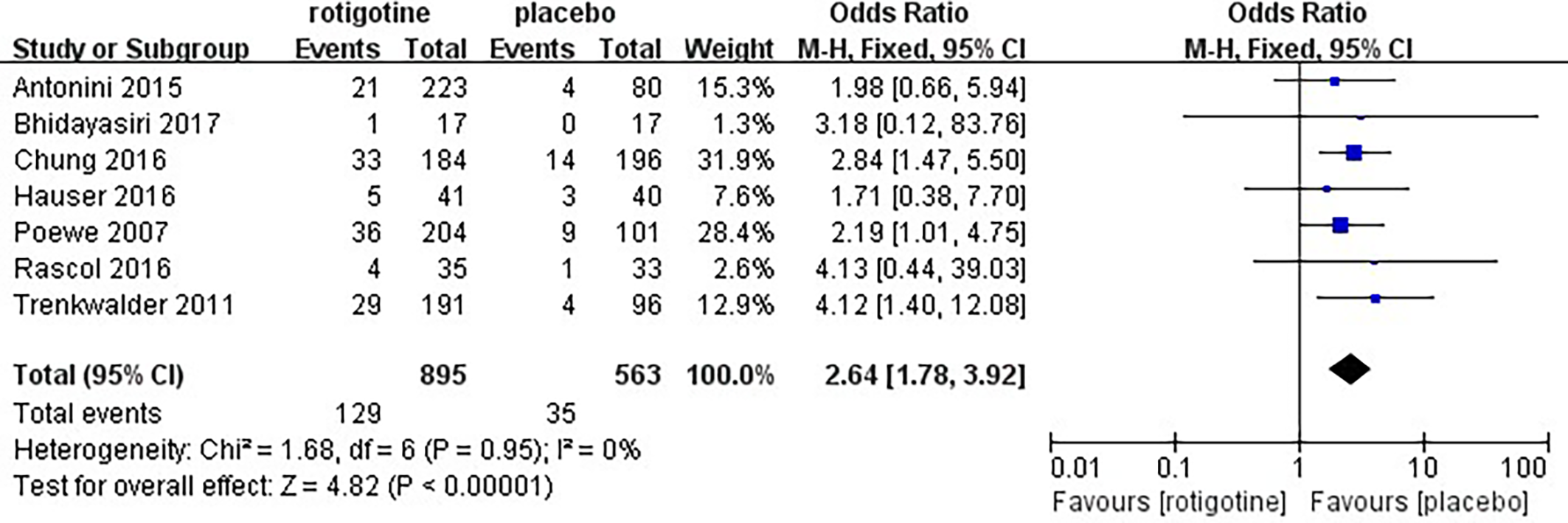


Forest plot of incidence of insomnia.


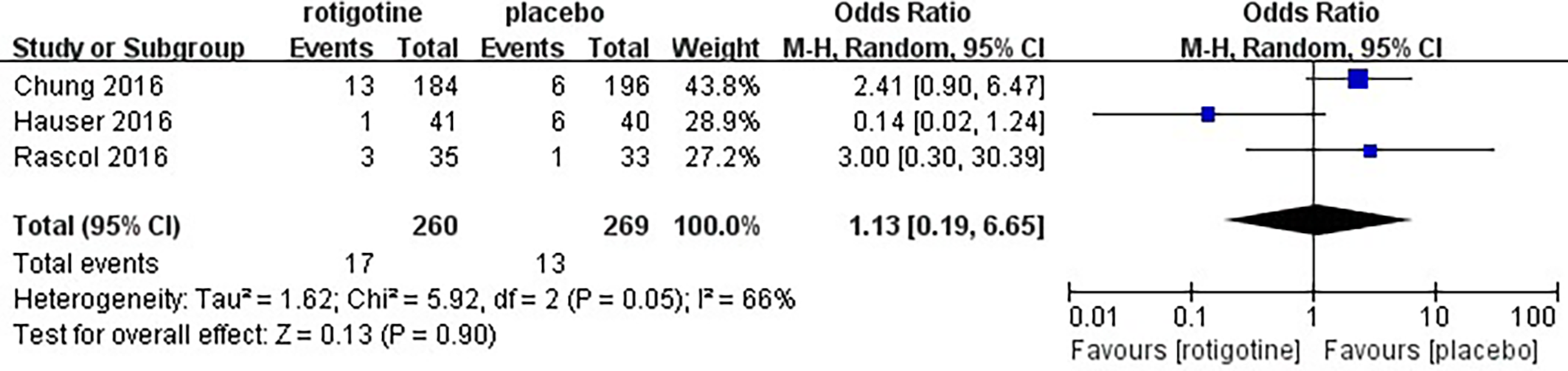


Sensitivity analysis: show the effect of rotigotine on PDQ-39 score in each studies.


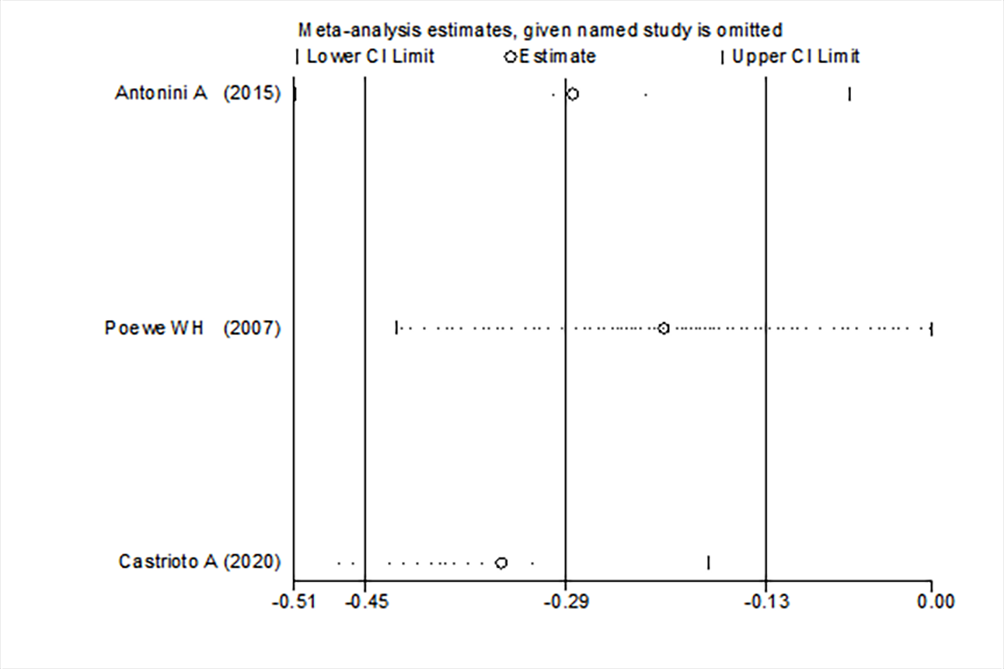


Sensitivity analysis: show the effect of rotigotine on PDQ score in each studies.


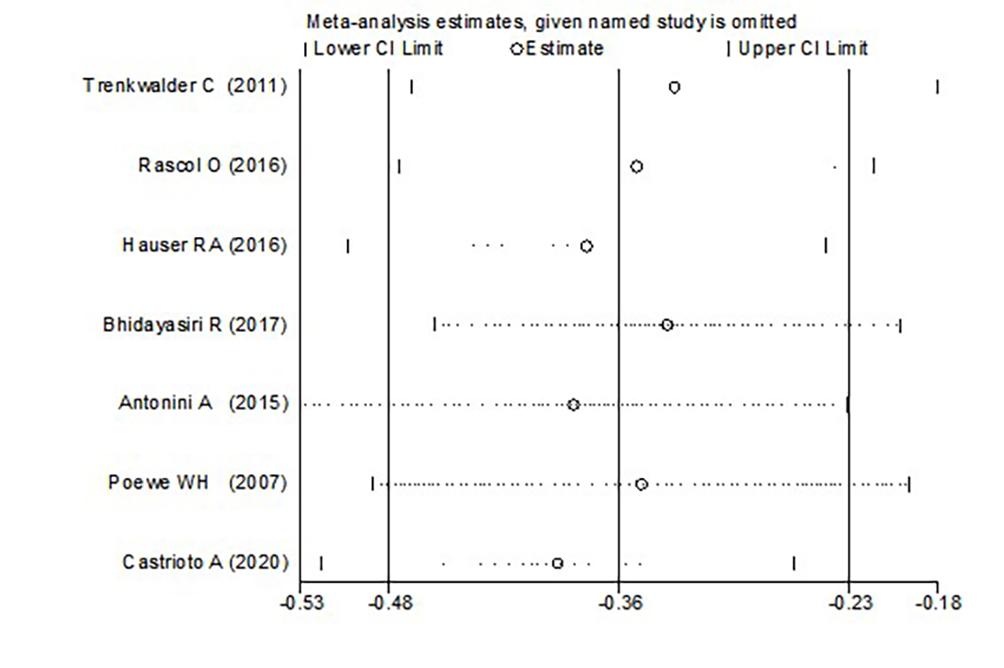

Supplement: Supplementary file 1 [file Data_Sheet_1.docx]
